# Injury burden in individuals aged 50 years or older in the Eastern Mediterranean region, 1990–2019: a systematic analysis from the Global Burden of Disease Study 2019

**DOI:** 10.1016/S2666-7568(22)00038-1

**Published:** 2022-04

**Authors:** Samar Al-Hajj, Sarah Farran, Abla M Sibai, Randah R Hamadeh, Vafa Rahimi-Movaghar, Rajaa Mohammad Al-Raddadi, Farideh Sadeghian, Zahra Ghodsi, Wael Alhajyaseeh, Niveen M Abu Rmeileh, Ali H Mokdad, GBD 2019 Collaborators

**Affiliations:** a Faculty of Health Sciences, American University of Beirut, Beirut, Lebanon; b Faculty of Medicine, American University of Beirut, Beirut, Lebanon; c College of Medicine and Medical Sciences, Arabian Gulf University, Manama, Bahrain; d Sina Trauma and Surgery Research Center, Tehran University of Medical Sciences, Tehran, Iran; e Department of Community Medicine, King Abdulaziz University, Jeddah, Saudi Arabia; f Center for Health Related Social and Behavioral Sciences Research, Shahroud University of Medical Sciences, Shahroud, Iran; g Qatar Transportation and Traffic Safety Center, and Department of Civil and Architectural Engineering, College of Engineering, Qatar University, Doha, Qatar; h Institute of Community and Public Health, Birzeit University, Birzeit, Palestine; i Institute for Health Metrics and Evaluation, University of Washington, Seattle, WA, USA

## Abstract

**Background:**

Injury poses a major threat to health and longevity in adults aged 50 years or older. The increased life expectancy in the Eastern Mediterranean region warrants a further understanding of the ageing population's inevitable changing health demands and challenges. We aimed to examine injury-related morbidity and mortality among adults aged 50 years or older in 22 Eastern Mediterranean countries.

**Methods:**

Drawing on data from the Global Burden of Diseases, Injuries, and Risk Factors Study (GBD) 2019, we categorised the population into adults aged 50–69 years and adults aged 70 years and older. We examined estimates for transport injuries, self-harm injuries, and unintentional injuries for both age groups, with sex differences reported, and analysed the percentage changes from 1990 to 2019. We reported injury-related mortality rates and disability-adjusted life-years (DALYs). The Socio-demographic Index (SDI) and the Healthcare Access and Quality (HAQ) Index were used to better understand the association of socioeconomic factors and health-care system performance, respectively, with injuries and health status in older people. Healthy life expectancy (HALE) was compared with injury-related deaths and DALYs and to the SDI and HAQ Index to understand the effect of injuries on healthy ageing. Finally, risk factors for injury deaths between 1990 and 2019 were assessed. 95% uncertainty intervals (UIs) are given for all estimates.

**Findings:**

Estimated injury mortality rates in the Eastern Mediterranean region exceeded the global rates in 2019, with higher injury mortality rates in males than in females for both age groups. Transport injuries were the leading cause of deaths in adults aged 50–69 years (43·0 [95% UI 31·0–51·8] per 100 000 population) and in adults aged 70 years or older (66·2 [52·5–75·5] per 100 000 population), closely followed by conflict and terrorism for both age groups (10·2 [9·3–11·3] deaths per 100 000 population for 50–69 years and 45·7 [41·5–50·3] deaths per 100 000 population for ≥70 years). The highest annual percentage change in mortality rates due to injury was observed in Afghanistan among people aged 70 years or older (400·4% increase; mortality rate 1109·7 [1017·7–1214·7] per 100 000 population). The leading cause of DALYs was transport injuries for people aged 50–69 years (1798·8 [1394·1–2116·0] per 100 000 population) and unintentional injuries for those aged 70 years or older (2013·2 [1682·2–2408·7] per 100 000 population). The estimates for HALE at 50 years and at 70 years in the Eastern Mediterranean region were lower than global estimates. Eastern Mediterranean countries with the lowest SDIs and HAQ Index values had high prevalence of injury DALYs and ranked the lowest for HALE at 50 years of age and HALE at 70 years. The leading injury mortality risk factors were occupational exposure in people aged 50–69 years and low bone mineral density in those aged 70 years or older.

**Interpretation:**

Injuries still pose a real threat to people aged 50 years or older living in the Eastern Mediterranean region, mainly due to transport and violence-related injuries. Dedicated efforts should be implemented to devise injury prevention strategies that are appropriate for older adults and cost-effective injury programmes tailored to the needs and resources of local health-care systems, and to curtail injury-associated risk and promote healthy ageing.

**Funding:**

Bill & Melinda Gates Foundation.

## Introduction

Injuries account for a significant portion of the global burden of disease and for more than 10% of all disability-adjusted life-years (DALYs).^1^ Known globally for being the leading cause of death among younger age groups, injuries represent one of the primary causes of death and disability among the older adult population.^2^, ^3^ As adults aged 50 years and older survive longer, their proportion in the population is projected to grow globally from 9% in 2016 to 15% in 2030.^4^, ^5^ Injury remains a threat to the wellbeing of people in this age group, and a public health concern that substantially drains health-care systems. Due to higher levels of frailty and the prevalence of comorbid non-communicable diseases,^6^ the ageing population has a higher risk of injuries, especially fractures and traumatic brain injuries resulting from fall-related injuries.^7^ These injuries compromise the overall health of older people and, in some cases, cause further complications resulting in hospital admission, extensive duration of hospital stays, poor functional outcomes at discharge, and even mortality.^8^ According to the Global Burden of Diseases, Injuries, and Risk Factors Study (GBD) 2019, more than 1·9 million individuals older than 50 years worldwide died as a result of injury in 2019, with many millions more sustaining debilitating non-fatal injuries.^2^ In addition to the health burden, injuries account for profound economic costs incurred on individuals and health-care systems.^9^

**Research in context**

**Evidence before this study**

The Eastern Mediterranean region faces a large injury-associated health-care burden, where unintentional injury is the leading cause of death and the second leading contributor to disability-adjusted life-years (DALYs). We searched Ovid MEDLINE for research articles published in English between Jan 1, 1946, and Oct 30, 2021, using medical subject heading terms “wounds and injuries” AND (“Afghanistan” OR “Bahrain” OR “Djibouti” OR “Egypt” OR “Iran” OR “Iraq” OR “Jordan OR “Kuwait” OR “Lebanon” OR “Libya” OR “Morocco” OR “Oman” OR “Pakistan” OR “Palestine” OR “Qatar” OR “Saudi Arabia” OR “Somalia” OR “Sudan” OR “Syria” OR “Tunisia” OR “United Arab Emirates” OR “Yemen”) AND (“middle aged (45 plus years)”) to limit the search to adults and older adults. Studies were eligible for review if they reported injuries in people aged 50 years or older in any of the countries in the Eastern Mediterranean region. Existing evidence suggests that research on the older population is scarce, with few studies addressing injury characteristics and underlying risk factors. A small number of studies had explored the injury status of the ageing population in the Eastern Mediterranean region, with particular focus on common trauma injury, including falls and traumatic brain injuries. In this study, we analysed estimates from the Global Burden of Disease, Injuries, and Risk Factors Study (GBD). GBD provides the latest estimates on the burden of injuries at the global, regional, and national levels. GBD 2019 data draw a comprehensive picture of injury estimates for people aged 50 years or older across Eastern Mediterranean countries from 1990 to 2019 by age, sex, year, country, and risk factors.

**Added value of this study**

This study provides the latest estimates on the burden of injury for adults aged 50–69 years and those aged 70 years or older in 22 countries in the Eastern Mediterranean region from 1990 to 2019. This first-time assessment of older adults’ injury burden is measured by injury-related mortality and DALY rates, Socio-demographic Index, Healthcare Access and Quality Index, healthy life expectancy, and risk factors for injury mortality. In addition to common trauma injuries related to transport and unintentional injuries, this study reveals the high prevalence of conflict and violence-related injuries and deaths among the ageing population in war-affected countries in the Eastern Mediterranean region. With potentially limited mobility and inability to escape war zones, people aged 70 years or older disproportionately suffer from war injuries that adversely affect their health conditions and their physical and mental wellbeing.

**Implications of all the available evidence**

Evidence from the study offers insights into the burden and leading types of injury and their associated disability and mortality among the ageing population in the Eastern Mediterranean region. Synthesised knowledge can serve to inform the implementation of injury prevention strategies that are appropriate for people aged 70 years or older, occupational injury prevention programmes, and policies that reduce injury burden and ensure healthy ageing.

Low-income and middle-income countries disproportionately carry a heavy toll of the global burden of injury.^2^ This burden is amplified among their vulnerable populations, particularly older adults because of their limited access to health-care services once they exit the labour market. The Eastern Mediterranean region has witnessed a growing population of people aged 50 years or older over the past two decades, resulting from increased life expectancy.^4^ This region is home to nearly 680 million people, and people aged 70 years or older constitute approximately 7·5% of its populations, ranging from 5% in the United Arab Emirates (UAE) to 10% in Lebanon.^4^ This percentage is expected to increase to 15% in the Eastern Mediterranean region by 2050 with advancement in life expectancy.^5^, ^10^ Compared with other regions, the Eastern Mediterranean region historically faces a large injury-associated morbidity and mortality burden, estimated at approximately 3·5 million life-years annually, mainly related to road transport and injuries associated with the regional wars and conflicts.^11^, ^12^ With the increase in life expectancy in the older population in the Eastern Mediterranean region, understanding the injury burden and the factors affecting this population's health and quality of life is of utmost relevance.

There is a paucity of studies examining the injury burden in adults aged 50 years and older, its characteristics, risk factors, and implications on local health-care systems in the Eastern Mediterranean region.^13^ Drawing on data from GBD 2019, we aim to assess the burden of injury mortality and morbidity among people aged 50–69 years and those aged 70 years or older in the Eastern Mediterranean region.

## Methods

### Overview

The GBD 2019 data were synthesised to assess injury burden for adults aged 50–69 and older adults aged at least 70 years in the Eastern Mediterranean region,^14^ which comprises 22 countries: Afghanistan, Bahrain, Djibouti, Egypt, Iran, Iraq, Jordan, Kuwait, Lebanon, Libya, Morocco, Oman, Pakistan, Palestine, Qatar, Saudi Arabia, Somalia, Sudan, Syria, Tunisia, the UAE, and Yemen. The two specified age groups were chosen based on the available GBD classification, whereby populations with different age distributions are statistically standardised to match those of a reference population and are grouped for an adequate representation of age-dependent diseases.^14^

Based on the methodology adopted in GBD 2019, injury morbidity and mortality were estimated from meta-regression models using data from multiple sources, including vital registration systems, verbal autopsies, surveys, censuses, and demographic surveillance sites, and code estimates to the International Classification of Diseases (ICD)-9 and ICD-10.^1^, ^2^, ^15^ The GBD 2019 is a collaborative multinational study that includes 369 diseases and injuries across 204 countries and territories between 1990 and 2019.^1^ The detailed methodology is available elsewhere.^16^

The GBD 2019 classifies injury into three categories: transport injuries (road-related injuries); self-harm and interpersonal violence (conflicts and terrorism, executions and police conflicts, physical violence by firearm, physical violence by sharp object, sexual violence, and physical violence by other means); and unintentional injuries (falls, drowning, fire, burns, poisonings, exposure to mechanical forces, animal contact, and foreign body ingestion). Injury causes and their matching classifications based on ICD-9 and ICD-10 codes along with injury diagnoses and reported causes of deaths and disabilities have been published in detail elsewhere.^2^ The codes to which injuries were mapped can be found in the appendix (pp 1–6).

To gain a deeper understanding of the association of socioeconomic factors and health-care system performance with older adults’ injury and health status, the Socio-demographic Index (SDI) and the Healthcare Access and Quality (HAQ) Index were used, which are composite interpretable indicators of, respectively, the relative development status of a country and the relative national level of health-care quality and access. The SDI ranges from 1 to 5 and uses country-level income per capita, average attained educational level (for the population older than 15 years old), and total fertility rate for females younger than 25 years. We categorised Eastern Mediterranean countries on the basis of SDI values and classified them into quintiles. The HAQ Index assesses mortality rates for preventable deaths from 32 causes, including injuries that can be avoidable in the presence of an effective health-care system, on a scale of 0 (worst) to 100 (best).^17^ The HAQ Index was compared with DALYs to assess whether there is a potential relationship between health-care quality and disability burden in the Eastern Mediterranean region. Moreover, healthy life expectancy (HALE) was reported for males and females separately. For any age, HALE is a single measure of a country's population health, which, unlike life expectancy, accounts for both fatal and non-fatal outcomes and is calculated based on life tables and per-capita estimates of years lived with disability (YLDs).^18^ The HALE values were compared with injury-related deaths and DALYs and to the country's SDI quintile and HAQ Index score to gain a comprehensive understanding of the effect of injury outcomes on achieving healthy ageing.

Ethics approval was deemed unnecessary as this study has accessed and analysed secondary data that are publicly available in the GBD Compare repository.^14^

### Statistical analysis

To estimate the burden of injury, the injury mortality and DALY rate estimates per 100 000 population were reported for the Eastern Mediterranean region overall and for each country in the region in 2019 and these estimates were compared with global trends. Separate estimates for males and females were reported for injury mortality in this region (appendix p 6). DALYs, defined as years of healthy life lost, are calculated based on an estimation of data for fatal and non-fatal injuries using the sum of years of life lost (YLLs) and YLDs for each injury cause, Eastern Mediterranean country, sex, year, and age group. YLLs is a measurement of premature injury mortality, estimated based on the sum of mortality data pertaining to each injury cause of death multiplied by standard expected individual lifespan at each age. YLDs are the years of life lived with any short-term or long-term health loss per person. YLDs are estimated using the number of cases of the disability, its average duration, and standardised disability weights for each health state for each injury cause, Eastern Mediterranean location, sex, year, and age^2^ (YLL and YLD results are detailed in the appendix pp 9, 12).

We assessed the percentage changes between 1990 and 2019 in injury mortality rates across Eastern Mediterranean countries by calculating the difference in mortality rate values between 1990 and 2019, divided by the 1990 rate value and multiplied by 100. The percentage change in HALE at 50 years (HALE-50) and HALE at 70 years (HALE-70) from 1990 to 2019 was calculated using the rate values for 1990 and 2019. To inform the prevalence of risks related to injury and to highlight factors that can be targeted by interventions, risk factors associated with the highest and lowest rates of injury death and DALYs across Eastern Mediterranean countries were reported, and included the following risks generated by the GBD: alcohol use, drug use, tobacco smoking, metabolic risks, low bone mineral density, intimate partner violence, behavioural risks, non-optimal temperature, and occupational risks. GBD uses six analytical steps to assess risk factors and 30 652 different data sources. Exposure levels are then summarised using the summary exposure value to allow comparison; a detailed method of this approach is available.^1^

The correlation between HAQ Index and DALYs for the two age groups was analysed using Pearson's correlation coefficient test, and significance was set at a p value of less than 0·05. The 95% uncertainty intervals (UIs) reported by the GBD for each estimate use the 2·5th and 97·5th percentile values of the distributions generated by the meta-regression models used.

### Role of the funding source

The funder of the study had no role in study design, data collection, data analysis, data interpretation, or writing of the report. All authors had full access to all of the data and the corresponding author had final responsibility to submit for publication.

## Results

**Figure 1:**
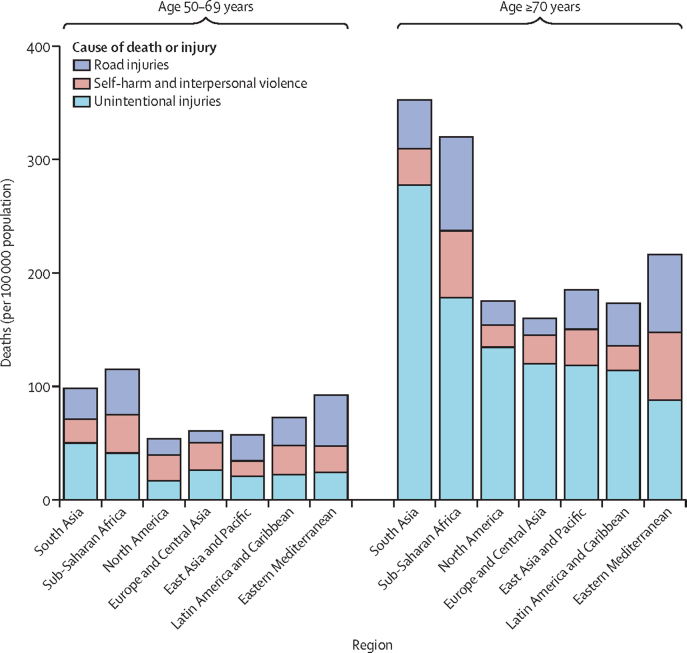
Injury death rates per 100 000 population

In 2019, the mortality rate due to injury in the Eastern Mediterranean region was 92·4 (95% UI 75·6–106·4) per 100 000 population for adults aged 50–69 years and 216·4 (190·7–239·3) per 100 000 population for those aged 70 years or older. These rates exceed the global estimates in 2019, with a rate of 71·4 (64·4–77·2) per 100 000 population at 50–69 years and 209·2 (182·4-227·3) per 100 000 population at 70 years or older (figure 1).^14^ Mortality rates were consistently higher in people aged 70 years or older than in those aged 50–69 years (Table 1, Table 2).

**Table 1:** Injury death rates in 1990, 2010, and 2019, and YLLs, YLDs, and DALYs by country for people aged 50–69 years in the Eastern Mediterranean region in 2019

	**Mortality rate per 100 000 population**				**YLL rate per 100 000 population, 2019**	**YLD rate per 100 000 population, 2019**	**DALY rate per 100 000 population, 2019**	**YLL/DALY, 2019**	**YLL/YLD**	
	1990	2010	2019	Percentage change, 1990–2019					1990	2019
Afghanistan	161·4 (126·8–206·3)	141·2 (110·4–176·3)	304·2 (272·4–340·3)	88·5%	9608·7 (8571·2–10 810·3)	4346·8 (2128·8–8672·3)	13 955·4 (11 147·2–18 758·3)	0·7	1·7	2·2
Bahrain	77·4 (64·3– 97·9)	39·1 (33·2–45·8)	29·3 (22·2–38·7)	−62·1%	916·4 (695·2–1214·3)	946·8 (670·6–1313·0)	1863·2 (1495·4–2281·2)	0·5	2·3	1·0
Djibouti	130·9 (95·1–176·6)	132·7 (89·2–185)	132·9 (97·3–180·6)	1·5%	4094·1 (2971·7–5597·8)	1324·3 (969·2–1728·6)	5418·4 (4249·2–7016·5)	0·8	3·1	3·1
Egypt	116·7 (76·7–138·6)	112·3 (73·2–136)	103·1 (60·4–145·3)	−11·6%	3168·8 (1839·2–4466·5)	869·2 (618·5–1182·9)	4038·0 (2690·7–5391·2)	0·8	3·9	3·6
Iran	275·9 (244·3–297·7)	73·6 (65·6–76·8)	62·9 (57·1–67·7)	−77·2%	1941·2 (1761·1–2087·2)	1275·2 (931·4–1705·0)	3216·4 (2816·5–3676·2)	0·6	4·7	1·5
Iraq	118·3 (90·9–156·4)	121·0 (92·9–153·0)	94·9 (68·7–124·3)	−19·7%	3043·8 (2201·1–4026·3)	2479·8 (1834·5–3296·4)	5523·6 (4486·3–6698·4)	0·6	1·2	1·2
Jordan	67·7 (56·7–83·7)	41·2 (35·6–50·1)	35·1 (27·9–44·8)	−48·1%	1095·0 (866·8–1397·1)	827·8 (585·1–1146·9)	1922·8 (1586·1–2337·0)	0·6	2·1	1·3
Kuwait	115·7 (106·6–124·1)	41·8 (39·4–44·0)	34·6 (27·3–43)	−70·1%	1099·6 (865·5–1372·8)	1109·6 (795·7–1530·0)	2209·3 (1818·2–2684·4)	0·5	3·1	1·0
Lebanon	88·0 (77·3–100·6)	34·3 (29·0–42·5)	28·5 (22·6–36·1)	−67·6%	866·6 (688·7–1107·3)	1739·7 (1077·8–2961·3)	2606·3 (1930·3–3819·3)	0·3	1·5	0·5
Libya	95·2 (60·8–123·1)	75·8 (51·4–86·2)	89·3 (63·1–114·4)	−6·2%	2818·4 (1976·6–3639·4)	1327·4 (969·7–1771·7)	4145·8 (3204·0–5046·7)	0·7	2·3	2·1
Morocco	108·8 (69·7–142·1)	89·3 (56·9–124·4)	87 (54·90–118·9)	−20·0%	2646·5 (1669·2–3636·6)	1197·2 (857·8–1629·9)	3843·7 (2795·6–4979·2)	0·7	2·5	2·2
Oman	252·3 (184·7–328·3)	193·4 (174·2–221·2)	100·5 (83·8–121·7)	−60·1%	3137·6 (2609·3–3779·7)	1187·4 (828·9–1647·7)	4324·9 (3706·5–5071·8)	0·7	5·2	2·6
Pakistan	71·3 (57·7–88·4)	75·8 (61·7–93·1)	60·0 (47·0–78·0)	−15·7%	1850·1 (1455·8–2405·4)	1364·2 (1001·0–1773·9)	3214·4 (2652·3–3891·0)	0·6	1·9	1·4
Qatar	161·1 (103·7–209·7)	86·9 (57·2–115·3)	61·8 (41·2–84)	−61·6%	1960·7 (1305·9–2695·2)	1274·5 (883·6–1778·6)	3235·2 (2441·1–4140·3)	0·6	3·0	1·5
Saudi Arabia	239·8 (166·1–317·4)	225·6 (150·7–260·3)	174 (114·7–221·3)	−27·4%	5596·7 (3704·6–7156·2)	2184·2 (1509·3–3031·3)	7780·9 (5777·1–9555·1)	0·7	3·5	2·6
Somalia	244 (183·1–325·5)	246·4 (179·7–327·2)	222·4 (161·8–303·7)	−8·8%	6700·9 (4850·8–9220·9)	1464·4 (1068·1–2004·7)	8165·4 (6220·5–10 663·0)	0·8	6·1	4·6
Sudan	154·5 (109·3–193·3)	103·2 (65·9–141·8)	85·2 (52·2–121·6)	−44·8%	2622·4 (1600·7–3765·8)	1174·0 (877·4–1543·2)	3796·5 (2752·2–4929·1)	0·7	4·5	2·2
Syria	52·5 (41·1–65·9)	38·8 (31·1–47·5)	139·1 (124·5–156·7)	165·1%	4106·1 (3669·2–4634·1)	1986·5 (1428·1–2695·4)	6092·6 (5293·3–7099·3)	0·7	1·6	2·1
Tunisia	77·1 (59·0–93·5)	59·8 (40·8–81·5)	54·1 (36·6–79·3)	−29·8%	1639·5 (1107·8–2404·1)	1010·1 (707·9–1400·4)	2649·6 (2049·0–3433·4)	0·6	2·1	1·6
United Arab Emirates	171·7 (112·4–232·0)	135·3 (89·6–184·0)	110·1 (65·1–162·6)	−35·8%	3618·3 (2152·7–5334·8)	1263·3 (902·5–1731·8)	4881·6 (3353·3–6624·8)	0·7	3·9	2·8
Yemen	168·4 (109·1–229·7)	124·5 (82·3–171·1)	153·4 (111·9–203·3)	−8·8%	4773·0 (3472·3–6348·3)	1422·2 (1055·8–1850·5)	6195·2 (4772·8–7898·9)	0·8	4·1	3·4

Data in parentheses are 95% uncertainty intervals. DALYs=disability-adjusted life-years. YLDs=years lived with disability. YLLs=years of life lost.

**Table 2:** Injury death rates in 1990, 2010, and 2019, and YLLs, YLDs, and DALYs by country for people aged 70 years or older in the Eastern Mediterranean region in 2019

	**Mortality rate per 100 000 population**				**YLL rate per 100 000 population, 2019**	**YLD rate per 100 000 population, 2019**	**DALY rate per 100 000 population, 2019**	**YLL/DALY, 2019**	**YLL/YLD**	
	1990	2010	2019	Percentage change, 1990–2019					1990	2019
Afghanistan	221·7 (177·4–277·5)	212·7 (172·4–260·8)	1109·7 (1017·7–1214·7)	400·4%	15 838·7 (14 531·9–17 322·8)	3476·2 (2134·7–5915·7)	19 314·9 (16 804·2–23 196·8)	0·8	1·3	4·6
Bahrain	108·8 (88·1–131·9)	124·7 (108·3–141·8)	108·8 (88·1–131·9)	−39·8%	1565·7 (1260·7–1924·1)	1092·8 (783·1–1500·7)	2658·6 (2206·3–3149·6)	0·6	2·5	1·4
Djibouti	335·5 (270·8–418·7)	349·2 (277·8–435·9)	348·9 (289·8–426·0)	4·0%	5281·4 (4361·2–6481·7)	1891·8 (1394·8–2470·3)	7173·2 (6144·5–8503·4)	0·7	2·8	2·8
Egypt	184·8 (134·1–219·9)	177·0 (127·3–211·7)	163·9 (113·4–216·8)	−11·3%	2493·5 (1685·9–3351·8)	1080·3 (766·8–1478·1)	3573·8 (2716·7–4504·5)	0·7	2·7	2·3
Iran	406·9 (365·9–438·6)	165·9 (150·0–185·5)	153·9 (137·3–175·4)	−62·2%	2019·1 (1822·3–2272·7)	1574·7 (1143·1–2089·4)	3593·9 (3116·8–4146·7)	0·6	2·4	1·3
Iraq	131·3 (104·5–165·6)	141·6 (115·0–172·4)	119·4 (95·4–145·3)	−9·1%	1736·7 (1387·3–2079·5)	2416·7 (1812·3–3107·2)	4153·5 (3480·9–4904·2)	0·4	0·4	0·7
Jordan	163·0 (137·9–195·4)	108·0 (93·5–129·3)	97·2 (80·9–120·3)	−40·3%	1356·1 (1113·8–1691·3)	1049·2 (743·1–1433·2)	2405·3 (2014·4–2888·2)	0·6	1·9	1·3
Kuwait	233·0 (204·7–252·6)	149·5 (128·6–162·9)	149·9 (118·8–178·1)	−35·6%	1778·0 (1421·7–2129·7)	1451·0 (1043·3–1961·9)	3229·0 (2668·3–3834·5)	0·6	2·3	1·2
Lebanon	161·9 (132·9–189·5)	95·0 (75·3–128·9)	87·2 (67·6–122·3)	−46·1%	1089·7 (869·9–1480·6)	1539·5 (1051·5–2339·0)	2629·2 (2085–3474·8)	0·4	1·1	0·7
Libya	187·0 (127·4–229·1)	156·7 (109·2–183·2)	174·4 (125·9–212·7)	−6·6%	2355·6 (1693·4–2894·9)	1585·1 (1144·4–2110·1)	3940·7 (3135·0–4737·2)	0·6	1·7	1·5
Morocco	237·6 (152·5–328·2)	219·5 (133·9–309·8)	221·6 (142·3–297·7)	−6·7%	3001·9 (1973·7–3985·9)	1572·4 (1128·9–2107·4)	4574·4 (3442·6–5771·0)	0·7	2·2	1·9
Oman	414·9 (306·5–520·1)	415·7 (362·6–495·0)	319·8 (268·2–390·4)	−22·9%	4596·0 (3815·9–5776·9)	1649·8 (1157·2–2267·1)	6245·8 (3353·3–7516·6)	0·7	3·5	2·8
Pakistan	192·7 (144·3–263·5)	194·2 (157·1–235·2)	167·5 (130·6–210·7)	−13·0%	2383·8 (1861·9–2978·0)	1592·4 (1168·0–2093·0)	3976·2 (3298·2–4736·6)	0·6	2	1·5
Qatar	312·1 (239·7–396·6)	301·6 (239·3–367·2)	219·0 (164·1–284·7)	−29·8%	3507·3 (2585·4–4610·2)	1543·4 (1078·4–2125·1)	5050·7 (4054·9–6256·6)	0·7	2·9	2·3
Saudi Arabia	389·5 (314·6–462·2)	334·3 (289·0–369·7)	274·0 (229·2–320·2)	−29·6%	4089·5 (3312·5–4820·6)	2931·6 (2055·1–4001·3)	7021·2 (2752·2–8374·1)	0·6	2·2	1·4
Somalia	505·6 (402·2–667·6)	466·2 (369·4–615·2)	432·5 (336·7–585·7)	−14·4%	6827 (5301·1–9247·1)	1762·0 (1304·9–2333·3)	8589·3 (6930·2–11 079·0)	0·8	4·4	3·9
Sudan	272·0 (194·8–335·5)	222·7 (152·0–279·7)	203·4 (136·5–259·3)	−25·2%	2898·6 (1899·4–3741·8)	1345·6 (992·8–1751·1)	4244·1 (3155·9–5171·2)	0·7	3·4	2·2
Syria	155·5 (113·6–192·4)	122·0 (92·7–143·7)	706·8 (642·7–774·7)	354·7%	9507·3 (8642·4–10 426·6)	3291·3 (2384·0–4422·8)	12 798·5 (11 410·0–14 359·2)	0·7	1·7	2·9
Tunisia	170·1 (137·5–203·6)	161·9 (122·1–208·0)	143·1 (108·1–188·8)	−15·8%	1931·2 (1427·1–2562·5)	1349·3 (954·6–1844·6)	3280·5 (2645·8–4062·8)	0·6	1·8	1·4
United Arab Emirates	347·3 (232·8–467·2)	391·6 (253·2–512·9)	212·3 (130·1–297·1)	−38·8%	3394·1 (2064·1–4860·3)	1440·6 (1019·9–1958·1)	4834·7 (3372·7–6464·3)	0·7	3·5	2·4
Yemen	220·2 (159·7–279·3)	206·0 (152·7–255·3)	249·1 (194·0–301·6)	13·1%	3724·7 (2887·3–4564·5)	1889·3 (1386·3–2453·4)	5614·1 (4600·5–6606·2)	0·7	2·6	0·7

Data in parentheses are 95% uncertainty intervals. DALYs=disability-adjusted life-years. YLDs=years lived with disability. YLLs=years of life lost.

For both age groups, males in the Eastern Mediterranean region and globally had higher injury mortality rates than females in 2019. The male-to-female ratio was nearly 2·2 for people aged 50–69 years and 1·5 for those aged 70 years or older.

The change in mortality rates due to injury from 1990 to 2019 was –0·013 for people aged 50–69 years and –0·002 for those aged 70 years or older (further details on male-to-female mortality rates are in the appendix pp 6, 10).

In 2019, the highest rates of injury deaths per 100 000 population for people aged 50–69 years were reported in Afghanistan at 304·2 (95% UI 272·4–340·3), followed by Somalia at 222·4 (161·8–303·7) and Saudi Arabia at 174·0 (114·7–221·3). The lowest reported rate for this age group was reported in Lebanon at the rate of 28·5 (22·6–36·1) deaths per 100 000 population, followed by Bahrain at 29·3 (22·2–38·7) and Kuwait at 34·6 (27·3–43·0) deaths per 100 000 population.

The highest mortality rate for people aged 70 years or older in 2019 was also observed in Afghanistan at 1109·7 (95% UI 1017·7–1214·7), followed by Syria at 706·8 (642·7–774·7) and Somalia at 432·5 (336·7–585·7) deaths per 100 000 population. The countries with the lowest rates for this age group were Lebanon at 87·2 (67·6–122·3), Jordan at 97·2 (80·9–120·3), and Bahrain at 108·8 (88·1–131·9) deaths per 100 000 population.

**Figure 2:**
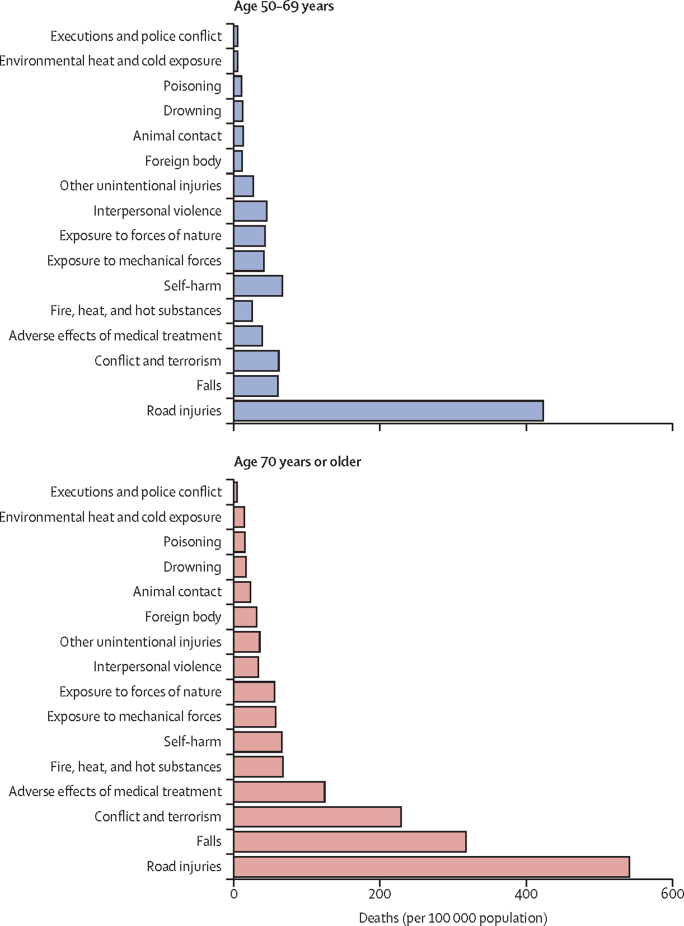
Rates of injury deaths per 100 000 population in 2019 in the Eastern Mediterranean region, classified by injury mechanisms and by age groups (50–69 years and ≥70 years)

**Figure 3:**
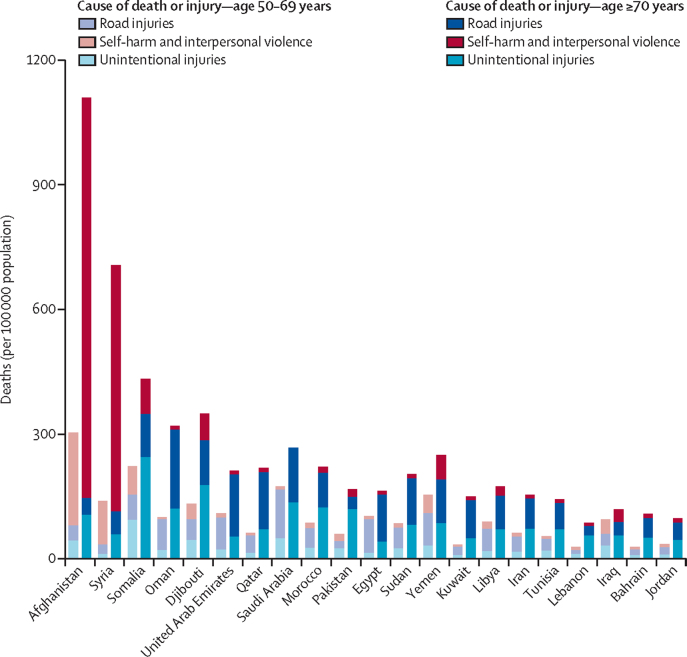
Distribution of injury death rates per 100 000 population among both age groups (50–69 years and ≥70 years) globally

In 2019, the leading cause of injury mortality in the Eastern Mediterranean region for both age groups was road injuries, with rates of 43·0 (95% UI 31·0–51·8) per 100 000 population for people aged 50–69 years and 66·2 (52·5–75·5) per 100 000 population for those aged 70 years or older (figure 2). The third leading cause of death was conflict and terrorism for both age groups (10·2 [9·3–11·3] deaths per 100 000 population for 50–69 years and 45·7 [41·5–50·3] deaths per 100 000 population for ≥70 years). The second leading cause of death was self-harm (7·5 [5·7–10·2] deaths per 100 000 population) for people aged 50–69 years and falls (42·5 [32·4–52·4] deaths per 100 000 population) for those aged 70 years or older (figure 3).

The highest percentage changes in injury deaths from 1990 to 2019 for people aged 50–69 years were in Afghanistan (88·5%) and Syria (165·1%), representing the highest increase. Iran had the largest decrease in injury deaths, with a percentage change of –77·2%, followed by Kuwait (–70·1%) and Lebanon (–67·6%).

For people aged 70 years or older, the country with the highest percentage in change in injury deaths between 1990 and 2019 was Afghanistan (400·4%), followed by Syria (354·7%) and Yemen (13·1%). In this age group, mortality rates per 100 000 population due to self-harm and violence ranged from 82·6 (95% UI 77·5–88·6) (2017) to 1120·3 (1050·9–1199·02) (2018) in Afghanistan, from 9·7 (7·7–11·8) (2010) to 3781·6 (3447·2–4148·7) (2014) in Syria, and from 14·5 (11·7–17·1) (2013) to 1519·3 (1380·8–1672·2) (2018) in Yemen. The highest percentage decreases during the same time period occurred in Iran (–62·2%), Lebanon (–46·1%), and Jordan (–40·3%).

In 2019, the injury-related DALY rate per 100 000 population in the Eastern Mediterranean region was 4305·5 (95% UI 3621·1–4950·4) for people aged 50–69 years and 4733·6 (4178·8–5371·3) for those aged 70 years or older.^14^ The global DALY rates were 3643·0 (3182·6–4173·5) per 100 000 population for people aged 50–69 years and 5090·8 (4344·8–5968·1) per 100 000 population for those aged 70 years or older).^14^ The leading cause of DALYs per 100 000 population reported in the Eastern Mediterranean region in 2019 was transport injuries for people aged 50–69 years (1798·8 [1394·1–2116·0]) and unintentional injuries for those aged 70 years or older (2013·2 [1682·2–2408·7]). The global leading causes of DALYs per 100 000 population were unintentional injuries for both age groups (1686·6 [1410·1–2030·7] for 50–69 years and 3510·8 [2931·8–4190·4] for 70 years or older).

In 2019, the country with the highest DALY rates per 100 000 population for people aged 50–69 years was Afghanistan (13 955·4 [95% UI 11 147·2–18 758·3]), followed by Somalia (8165·4 [6220·5–10 663·0]) and Saudi Arabia (7781·0 [5777·1–9555·5]) despite its high SDI (0·81). The lowest DALY rates per 100 000 population were in Bahrain (1863·2 [1495·4–2281·2]), Jordan (1922·8 [1586·1–2337·0]), and Kuwait (2209·3 [1818·2–2684·4]).

For people aged 70 years or older, the highest DALY rates per 100 000 population in 2019 were in Afghanistan (19 314·9 [95% UI 16 804·2–23 196·8]), followed by Syria (12 798·5 [11 410·0–14 359·2]) and Somalia (8589·3 [6930·2–11 079·0]). For this age group, the lowest DALY rates per 100 000 population were for Jordan (2405·3 [2014·4–2888·2]), followed by Lebanon (2629·2 [2085·0–3474·8]), and Bahrain (2658·5 [2206·3–3149·6]).

The SDI varied extensively among Eastern Mediterranean countries in 2019, from 0·08 in Somalia to 0·88 in the UAE. High injury mortality rates per 100 000 population among people aged 50–69 years were reported in countries with a high SDI such as UAE (110·1 [95% UI 65·1–162·6]), Qatar (61·8 [41·2–84·0]), and Kuwait (34·6 [27·3–43·0]; table 1). High adult injury mortality rates were reported in these Eastern Mediterranean countries with high SDIs compared with countries with similar SDIs, such as South Korea (67·3 [51·0–74·6]), France (53·6 [51·0–56·6]), and Cyprus (36·8 [33·0–41·0]).^14^ For people aged 70 years or older, the injury mortality rate per 100 000 population was 212·3 (130·1–297·1) for the UAE, 150·0 (118·8–178·2) for Kuwait, and 219·0 (164·1–284·7) for Qatar.

The most common causes of death in people aged 50–69 years in Eastern Mediterranean countries with the lowest SDIs were self-harm and interpersonal violence in Afghanistan (224·5 [95% UI 205·2–247·3] deaths per 100 000 population), unintentional injuries in Somalia (94·3 [62·8– 168·7] deaths per 100 000 population), and road injuries in Yemen (78·3 [47·4–112·1] deaths per 100 000 population). Self-harm and interpersonal violence were also the most common causes of death among people aged 70 years or older in Afghanistan (963·1 [876·2–1061·1] deaths per 100 000 population), followed by unintentional injuries in Somalia (245·3 [180·0–394·0] deaths per 100 000 population), and road injuries in Yemen (103·6 [70·6–136·5] deaths per 100 000 population).

Kuwait (82·6 [95% UI 81·2–83·9]), Qatar (81·72 [79·9–862·5]), and Lebanon (81·2 [79·8–82·3]) had the highest HAQ Index for people aged 50–69 years, aligning with the global HAQ Index of 73·8.^19^ Somalia (16·1 [15·1–17·2]), Afghanistan (26·9 [25·4–28·4]), and Pakistan (35·6 [34·1–37·1]) had the lowest HAQ Index. HAQ Index values were inversely correlated with injury-related DALY rates reported in these countries, whereby countries with the lowest HAQ Index values had higher DALY rates due to injuries (Pearson's *r*=–0·63, p=0·002 for people aged 50–69 years and Pearson's *r*=–0·33, p=1·49 for those aged 70 years or older).

In the Eastern Mediterranean region in 2019, the mean HALE-50 was 20·6 (95% UI 19·0–22·3) for both sexes combined, 20·4 (18·8–22·0) for males, and 20·9 (19·1–22·7) for females. These rates are lower than the global HALE-50 rate of 22·8 (21·1–24·4) for both sexes, 21·8 (20·2–23·3) for males, and 23·9 (21·9–25·6) for females.^14^ The Eastern Mediterranean countries with the highest-ranking HALE-50 for both sexes were Kuwait (25·7 [23·5–27·7]), Jordan (24·2 [–22·2-26·1]), and Iran (23·7 [21·8-25·5]). Those countries with the lowest HALE-50 ranked among the highest in injury-related death and DALY rates and the lowest in SDIs and HAQ Index values, including Afghanistan (16·7, 26·9 [25·4–28·4]), Somalia (16·9, 16·1 [15·1–17·2]), and Pakistan (19·14, 35·6 [34·1–37·1]). Overall, there was an increase in HALE-50 for all Eastern Mediterranean countries, with the highest percentage change reported in Jordan and Iran (both 16%) and the lowest reported in Libya (<0·01%).

HALE-70 for the Eastern Mediterranean region was 8·5 (95% UI 7·7–9·4) for both sexes, 8·5 (7·6–9·4) for males, and 8·6 (7·7–9·5) for females. The global HALE-70 in 2019 was 9·9 (8·9–10·8) for both sexes, 9·2 (8·3–10·1) for males, and 10·5 (9·3–11·5) for females.^14^ Kuwait (11·4 [10·1–12·7]), Saudi Arabia (9·0 [7·9–10·1]), and the UAE (8·4 [7·3–9·5]) had the highest HALE-70 for both sexes in the Eastern Mediterranean region in 2019. These three countries also ranked among those countries with the highest regional SDIs in 2019. HAQ Index values in 2019 were 82·6 (81·2–83·9) for Kuwait, 74·3 (72·8–75·8) for Saudi Arabia, and 61·2 (59·5–62·7) for the UAE. The lowest HALE-70 was reported in Afghanistan (6·7 [5·8–7·7]), Somalia (7·2 [6·1–8·2]), and Oman (7·5 [6·7–8·4]). Afghanistan and Somalia ranked among the highest in injury-related death and DALY rates and were in the lowest SDI quintiles and had the lowest HAQ Index values in 2019. For Oman, the SDI was 0·78 and HAQ Index was 76·1 (74·7–77·6). There was an increase in HALE-70 between 1990 and 2019 for all Eastern Mediterranean countries except for Syria (–0·01%); the highest percentage increase in HALE-70 was reported in Bahrain (29%), the UAE (25%), and Jordan (25%).

The leading injury mortality risk factors were occupational exposure in people aged 50–69 years and low bone mineral density in those aged 70 years or older. The risk factor for injury-related deaths in adults was mostly linked to occupational exposure in Somalia, with a mortality rate per 100 000 population of 29·1 (95% UI 23·9–35·4), followed by low bone mineral density in Saudi Arabia 25·3 (15·6–33·0) and Somalia 16·1 (11·6–22·3). Similarly, the risk factor associated with the largest injury DALY rate per 100 000 population was occupational exposure in Somalia (1161·0 [937·0–1429·4]), followed by low bone mineral density, which was highest in Saudi Arabia (1149·3 [815·3–1442·0]) and Somalia (655·7 [507·3–847·4]).

The largest risk factor associated with increased rates of injury death in people aged 70 years or older was low bone mineral density, mostly in Saudi Arabia (82·4 [95% UI 67·0–97·4]), followed by Oman (80·8 [65·7–95·9]) and Djibouti (66·6 [54·8–82·4]). The highest DALY rates per 100 000 population were also linked to low bone mineral density and were highest in the same countries (2167·0 [1744·8–2610·8] in Saudi Arabia, 1618·0 [1337·1–1932·6] in Oman, and 1437·2 [1206·3–1718·1] in Djibouti).

## Discussion

This study presents, to our knowledge, the first comprehensive estimates of the injury burden in people aged 50 years or older in the Eastern Mediterranean region from 1990 to 2019. Because most countries in this region have invested insufficient resources in injury prevention among the older population,^13^, ^20^ this study offers insights into the importance of reshaping the infrastructure of existing health-care systems in the region to improve health-care provision and access for the growing ageing population.

Compared with global rates, the observed rates of injury morbidity and mortality are relatively high in many Eastern Mediterranean countries regardless of their SDIs, signalling an alarming threat to the health and longevity of older people in the region. The rates of injury-related deaths and DALYs are noticeably higher in the Eastern Mediterranean region, up to three times higher than in industrialised countries such as Italy, Switzerland, and Denmark with similar SDIs,^14^, ^21^, ^22^ or in countries of cultural similarities or geographical proximity, such as Turkey. Countries in the Eastern Mediterranean with high SDIs reported higher injury mortality rates for people aged 50–69 years than countries with similar SDIs (eg, South Korea, France, and Cyprus). These high injury death and disability rates reflect the weaknesses and fragility of local health-care systems across the Eastern Mediterranean region, evidenced by their limited resources, scarce provision of vital emergency services, and the absence of rehabilitation services to injury survivors.

Road traffic and conflict and terrorism-related injuries constitute the leading causes of fatality among both adults aged 50–69 years and those aged 70 years and older in Eastern Mediterranean countries. Road traffic injury remains a major public health problem across all age groups in the region.^11^, ^23^ This trend is primarily associated with excess speed and the absence of safe road infrastructure and of law enforcement in high SDI countries (ie, Oman, the UAE, Saudi Arabia, and Qatar^24^, ^25^), and with poorly maintained vehicles in sanctioned and low SDI countries in the region.^26^ These findings underscore the urgent need to implement targeted safety interventions together with strict enforcements of transport regulations. Contrary to global trends of the leading causes of injury in older adults, conflicts and terrorism threaten the health of the older population in the Eastern Mediterranean region, particularly in countries with high conflicts, such as Syria, Afghanistan, and Yemen. Mortality rates related to conflicts and terrorism increased substantially among these countries, which explains the increase in their average injury-related mortality rates across the years from the years 1990 to 2019. These worrying estimates call for international laws to protect the vulnerable ageing population and strongly advocate for preventing exposure of older people to conflict and terrorism-related harms.

The observed estimates for the SDI and HAQ Index represented key indicators of injury characteristics. Eastern Mediterranean countries with a low SDI consistently reported a low HAQ Index, which was manifested in the high prevalence of injury DALY rates. This demonstrated the evident association between SDI, HAQ Index, HALE-50, and HALE-70. Comparing the HALE of countries to their injury-related deaths, DALYs, SDI, and HAQ Index values strongly suggests that the reduction in injury morbidity and mortality achieves healthy ageing and primarily leads to gains in HALE. The leading risk factor for injuries across Eastern Mediterranean countries was low bone mineral density, emphasising its significant association with injury-related disabilities.^27^ In light of these findings, Eastern Mediterranean countries should strive to develop and implement country-level injury prevention programmes and interventions that reflect their HAQ index levels in an attempt to provide sustainable and healthy ageing to their older adult populations by improving quality and access to health-care services while reducing health costs on individuals and health-care systems. Governments should be prepared to effectively accommodate their ageing population and the concomitant rise in its needs through implemented policies and programmes.

Eastern Mediterranean countries should prioritise national injury prevention programmes tailored towards implementing injury education and preventive strategies to reduce the most common injuries (eg, fall-related traumatic brain injuries), in addition to mental disorders and non-communicable diseases, which exacerbate injury risk among older adults.^7^ These strategies should include education and awareness for older adults and their caregivers and occupational injury prevention. Moreover, rigorous governmental action should be adopted to promote routine bone density screening,^20^ frailty assessment in older adults, and close monitoring of medications and vitamin deficiencies, exercise, and nutritional optimisation for the ageing population. It is crucial for local governments to integrate tailored and culturally sensitive injury interventions depending on existing infrastructure, capacities, and resources^28^ for effective and sustainable injury solutions.

As most injury-related deaths are potentially preventable, improving trauma care and access to health-care and post-injury rehabilitation services could save thousands of lives annually.^29^ The ageing population is known to account for the highest share of health-care expenditure and long-term care costs.^9^ Reducing injuries in older adults will alleviate the financial burden on families and local health-care systems. In most Eastern Mediterranean countries, regardless of their SDIs, care facilities for older adults are near absent, particularly with the embedded cultural and religious practices of familial care for older members.^30^ Often, family members assume the caregiver role and carry the burden of caring for the injured older adults. National strategies should therefore ensure the provision of affordable geriatric health services to alleviate the burden on older people and their caregivers.

This study has some limitations. First, this is a secondary data analysis with inherited limitations from the underlying dataset, hence hindering a full understanding of the causes of injury due to the lack of additional information on contributing risk factors to higher rates of injuries. Second, the large regional burden of conflicts and terrorism-related injuries impedes the generalisability and the implications of the findings to other regions. Third, a large number of Eastern Mediterranean countries lack injury surveillance programmes, vital statistics, health information systems, and trauma registries, which might limit the representativeness of the available country-specific data. Fourth, war-affected Eastern Mediterranean countries are prone to the absence of reliable and well documented health data, which, together with the political misinformation in some of these countries, presents an additional obstacle to collecting accurate and representative data.

The injury burden among older people across the Eastern Mediterranean region, including in high SDI countries, exceeded global estimates. Road traffic injuries and conflicts and terrorism-related injuries represent the primary causes of fatal injuries among this target population. Synthesised knowledge can serve to implement injury prevention strategies appropriate for older people, occupational injury prevention programmes, and policies that reduce the burden of injury and ensure healthy ageing. This study warrants further investigation to better understand the characteristics and risk factors associated with this vulnerable population and to reduce injuries causing deaths and long-term disabilities.

## Data sharing

The datasets generated or analysed during the current study are available in the GBD Compare repository.

## Declaration of interests

We declare no competing interests.
