# Correction to *Lancet Healthy Longev* 2022; 3: e253–62

**DOI:** 10.1016/S2666-7568(22)00130-1

**Published:** 2022-06

*Al-Hajj S, Farran S, Sibai AM, et al. Injury burden in individuals aged 50 years or older in the Eastern Mediterranean region, 1990–2019: a systematic analysis from the Global Burden of Disease Study 2019.* Lancet Healthy Longev 2022; 3: *e253–62*—In this Article, the spelling of author Wael Alhajyaseen's name was incorrect. This correction has been made as of June 8, 2022.

