# Supplementary material for: Injury burden in individuals aged 50 years or older in the Eastern Mediterranean region, 1990–2019: a systematic analysis from the Global Burden of Disease Study 2019
Source: Lancet Healthy Longev. 2022 Apr;3(4):e253–62. doi: 10.1016/S2666-7568(22)00038-1 (PMC8979829; doi:10.1016/S2666-7568(22)00038-1)
Supplement: Supplementary appendix [file mmc1.pdf]

THE LANCET
Healthy Longevity

Supplementary appendix

This appendix formed part of the original submission and has been peer reviewed. We post it as supplied by the authors.

Supplement to: Al-Hajj S, Farran S, Sibai AM, et al. Injury burden in individuals aged 50 years or older in the Eastern Mediterranean region, 1990–2019: a systematic analysis from the Global Burden of Disease Study 2019. *Lancet Healthy Longev* 2022; **3:** e253–62.

Appendix

List of International Classification of Diseases (ICD) codes mapped to the Global Burden of Disease cause list

	Cause	ICD10	ICD10 Used in Hospital/Claims Analyses	ICD9	ICD9 Used in Hospital/Claims Analyses
315	Transport injuries	V00-V86.99, V87.2-V87.3, V88.2-V88.3, V90-V98.8	V00-V98.8	E800-E800.3, E801-E801.3, E802-E802.3, E803-E803.3, E804-E804.3, E805-E805.3, E806-E806.3, E807-E807.3, E810.0-E810.7, E811.0-E811.7, E812.0-E812.7, E813.0-E813.7, E814.0-E814.7, E815.0-E815.7, E816.0-E816.7, E817.0-E817.7, E818.0-E818.7, E819.0-E819.7, E820.0-E820.7, E821.0-E821.7, E822.0-E822.7, E823.0-E823.7, E824.0-E824.7, E825.0-E825.7, E826.0-E826.4, E827.0-E827.4, E828.0-E828.4, E829.0-E829.4, E830-E838.9, E840-E849.9, E929.1, V03, V07.8-V07.9, V13, V13.8, V13.9, V15.2, V15.3, V15.9, V19, V42, V42.8, V42.9-V43, V47-V47.1	E80.0-E84.99, E92.91
316	Road injuries	V01-V04.99, V06-V80.929, V82-V82.9, V87.2-V87.3	V01-V04.99, V06-V80.929, V82-V82.9, V87.2-V87.3	E800.3, E801.3, E802.3, E803.3, E804.3, E805.3, E806.3, E807.3, E810.0-E810.6, E811.0-E811.7, E812.0-E812.7, E813.0-E813.7, E814.0-E814.7, E815.0-E815.7, E816.0-E816.7, E817.0-E817.7, E818.0-E818.7, E819.0-E819.7, E820.0-E820.6, E821.0-E821.6, E822.0-E822.7, E823.0-E823.7, E824.0-E824.7, E825.0-E825.7, E826.0-E826.1, E826.3-E826.4, E827.0, E827.3-E827.4, E828.0, E828.4, E829.0-E829.4, V03, V07.8-	E80.03, E80.13, E80.23, E80.33, E80.43, E80.53, E80.63, E80.73-E81.06, E81.10-E82.06, E82.10-E82.16, E82.20-E82.61, E82.63-E82.70, E82.73-E82.80, E82.84-E82.94

				V07.9, V13, V13.8, V13.9, V15.2, V15.3, V15.9, V19, V42, V42.8, V42.9-V43, V47-V47.1	
317	Pedestrian road injuries	V01-V04.99, V06-V09.9	V01-V04.99, V06-V09.9	E811.7, E812.7, E813.7, E814.7, E815.7, E816.7, E817.7, E818.7, E819.7, E822.7, E823.7, E824.7, E825.7, E826.0, E827.0, E828.0, E829.0, V03, V07.8-V07.9	E81.17, E81.27, E81.37, E81.47, E81.57, E81.67, E81.77, E81.87, E81.97, E82.27, E82.37, E82.47, E82.57-E82.60, E82.70, E82.80, E82.90
318	Cyclist road injuries	V10-V19.9	V10-V19.9	E800.3, E801.3, E802.3, E803.3, E804.3, E805.3, E806.3, E807.3, E810.6, E811.6, E812.6, E813.6, E814.6, E815.6, E816.6, E817.6, E818.6, E819.6, E820.6, E821.6, E822.6, E823.6, E824.6, E825.6, E826.1, V13, V13.8, V13.9, V15.2, V15.3, V15.9, V19	E80.03, E80.13, E80.23, E80.33, E80.43, E80.53, E80.63, E80.73, E81.06, E81.16, E81.26, E81.36, E81.46, E81.56, E81.66, E81.76, E81.86, E81.96, E82.06, E82.16, E82.26, E82.36, E82.46, E82.56, E82.61
319	Motorcyclist road injuries	V20-V29.9	V20-V29.9	E810.2-E810.3, E811.2-E811.3, E812.2-E812.3, E813.2-E813.3, E814.2-E814.3, E815.2-E815.3, E816.2-E816.3, E817.2-E817.3, E818.2-E818.3, E819.2-E819.3, E820.2-E820.3, E821.2-E821.3, E822.2-E822.3, E823.2-E823.3, E824.2-E824.3, E825.2-E825.3	E81.02-E81.03, E81.12-E81.13, E81.22-E81.23, E81.32-E81.33, E81.42-E81.43, E81.52-E81.53, E81.62-E81.63, E81.72-E81.73, E81.82-E81.83, E81.92-E81.93, E82.02-E82.03, E82.12-E82.13, E82.22-E82.23, E82.32-E82.33, E82.42-E82.43, E82.52-E82.53
320	Motor vehicle road injuries	V30-V79.9, V87.2-V87.3	V30-V79.9, V87.2-V87.3	E810.0-E810.1, E811.0-E811.1, E812.0-E812.1, E813.0-E813.1, E814.0-E814.1, E815.0-E815.1, E816.0-E816.1, E817.0-E817.1, E818.0-E818.1, E819.0-E819.1, E820.0-E820.1, E821.0-E821.1, E822.0-E822.1, E823.0-E823.1, E824.0-E824.1, E825.0-E825.1, V42, V42.8, V42.9-V43, V47-V47.1	E81.00-E81.01, E81.10-E81.11, E81.20-E81.21, E81.30-E81.31, E81.40-E81.41, E81.50-E81.51, E81.60-E81.61, E81.70-E81.71, E81.80-E81.81, E81.90-E81.91, E82.00-E82.01, E82.10-E82.11, E82.20-E82.21, E82.30-E82.31, E82.40-E82.41, E82.50-E82.51

321	Other road injuries	V80-V80.929, V82-V82.9	V80-V80.929, V82-V82.9	E810.4-E810.5, E811.4-E811.5, E812.4-E812.5, E813.4-E813.5, E814.4-E814.5, E815.4-E815.5, E816.4-E816.5, E817.4-E817.5, E818.4-E818.5, E819.4-E819.5, E820.4-E820.5, E821.4-E821.5, E822.4-E822.5, E823.4-E823.5, E824.4-E824.5, E825.4-E825.5, E826.3-E826.4, E827.3-E827.4, E828.4, E829.4	E81.04-E81.05, E81.14-E81.15, E81.24-E81.25, E81.34-E81.35, E81.44-E81.45, E81.54-E81.55, E81.64-E81.65, E81.74-E81.75, E81.84-E81.85, E81.94-E81.95, E82.04-E82.05, E82.14-E82.15, E82.24-E82.25, E82.34-E82.35, E82.44-E82.45, E82.54-E82.55, E82.63-E82.64, E82.73-E82.74, E82.84, E82.94
322	Other transport injuries	V00-V00.898, V05-V05.99, V81-V81.9, V83-V86.99, V88.2-V88.3, V90-V98.8	V00-V00.898, V05-V05.99, V81-V81.9, V83-V86.99, V88.2-V98.8	E800-E800.2, E801-E801.2, E802-E802.2, E803-E803.2, E804-E804.2, E805-E805.2, E806-E806.2, E807-E807.2, E810.7, E820.7, E821.7, E826.2, E827.2, E828.2, E830-E838.9, E840-E849.9, E929.1	E80.0-E80.02, E80.1-E80.12, E80.2-E80.22, E80.3-E80.32, E80.4-E80.42, E80.5-E80.52, E80.6-E80.62, E80.7-E80.72, E81.07, E82.07, E82.17, E82.62, E82.72, E82.82, E83.0-E84.99, E92.91
323	Unintentional injuries	D69.5-D69.59, D70.1-D70.2, D78-D78.89, D89.81-D89.813, E03.2, E06.4, E09-E09.9, E16.0, E23.1, E24.2, E27.3, E36-E36.8, E66.1, E86.02-E87.99, E89-E89.9, G21.0-G21.19, G24.0-G24.09, G25.1, G25.4, G25.6-G25.79, G62.0, G72.0, G93.7, G96.0, G96.11, G97-G97.9, H02.81-H02.819, H05.33-H05.339, H05.42-H05.53, H44.6-H44.799, H59-H59.89, H91.0-H91.09, H95-H95.9, I95.2-I95.81, I97-I97.9, J70-J70.5, J95-J95.9, K08.5-K08.59, K43-K43.9, K52.0, K62.7, K68.11, K91-K91.9, K94-K95.89, L23.3, L27.0-L27.1, L55-L55.9, L56.0-L56.1, L58-L58.9, L64.0, L76-L76.82, M10.2-M10.29, M60.2-M60.28, M87.1-M87.19, M96-M96.9, N14-N14.4, N30.4-N30.41, N46.021-N46.121, N52.2-	D69.5-D69.59, D70.1-D70.2, D78-D78.89, D89.81-D89.813, E03.2, E06.4, E09-E09.9, E16.0, E23.1, E24.2, E27.3, E36-E36.8, E66.1, E89-E89.9, G21.0-G25.79, G62.0-G97.9, H02.81-H02.819, H05.33-H05.339, H05.42-H05.53, H44.6-H44.799, H59-H59.89, H91.0-H91.09, H95-H95.9, I95.2-I97.9, J70-J70.5, J95-J95.9, K08.5-K08.59, K43-K43.9, K52.0, K62.7, K68.11, K91-K91.9, K94-K95.89, L23.3, L27.0-L27.1, L55-L55.9, L56.0-L56.1, L58-L58.9, L64.0, L76-L76.82, M10.2-M10.29, M60.2-M60.28, M87.1-M87.19, M96-M96.9, N14-N14.4, N30.4-N30.41, N46.021-N46.121, N52.2-N52.39, N65-N65.1, N99-N99.9, P93-P93.8, P96.2-P96.5, R50.2-R50.83, W00-X58.9, Y40-Y84.9, Y88-Y88.3, Z21.0,	244.0-244.1, 244.3, 251.3, 253.7, 279.5-279.53, 331.81, 333.92, 349-349.9, 357.6, 359.24, 360.5-360.69, 374.86, 376.6, 379.6-379.63, 440.3-440.32, 457.0, 458.2-458.29, 518.6-518.7, 519.0-519.1, 525.6-525.79, 526.62-526.63, 530.86-530.87, 536.4-536.49, 539-539.9, 551.2-551.29, 552.2-552.29, 553.2-553.29, 564.2-564.4, 569.6-569.8, 579.3, 595.82, 596.81-596.83, 598.2, 612-612.1, 709.4, 770.1-770.18, 779.4-779.5, 780.62-780.66, 995.89, E850.3-E858.99, E862-E869.99, E870-E876.9, E878-E879.9, E880-E886.99, E888-E928.89, E929.2-E929.5, E930-E949.9, V44-V45, V45.2-V45.4, V45.7, V45.77, V45.79-V45.8, V45.87-V45.89	244.0-244.1, 244.3, 251.3, 253.7, 279.5-279.53, 331.81, 333.92, 349-349.9, 357.6-359.24, 360.5-360.69, 374.86, 376.6, 379.6-379.63, 440.3-440.32, 457.0, 458.2-458.29, 518.6-519.1, 525.6-525.79, 526.62-526.63, 530.86-530.87, 536.4-536.49, 539-539.9, 551.2-551.29, 552.2-552.29, 553.2-553.29, 564.2-564.4, 569.6-569.8, 579.3, 595.82, 596.81-596.83, 598.2, 612-612.1, 709.4, 770.1-770.18, 779.4-780.66, 995.89, E85.6-E92.889, E92.93-E94.99, V44-V45.89

		N52.39, N65-N65.1, N99-N99.9, P93-P93.8, P96.2, P96.5, R50.2-R50.83, W00-W46.2, W49-W62.9, W64-W70.9, W73-W81.9, W83-W94.9, W97.9, W99-X06.9, X08-X44.9, X46-X58.9, Y10-Y14.9, Y16-Y19.9, Y40-Y84.9, Y88-Y88.3, Z21.0, Z42-Z43.0, Z43.8-Z43.9, Z48-Z48.9, Z51-Z51.9, Z88-Z88.9, Z92-Z94.0, Z94.6, Z94.8-Z94.9, Z96-Z96.49, Z96.6-Z97.2, Z97.8-Z99.12, Z99.3-Z99.9	Z42-Z51.9, Z88-Z94.0, Z94.6-Z99.9		
324	Falls	W00-W19.9	W00-W19.9	E880-E886.99, E888-E888.9, E929.3	E88.0-E88.89, E92.93
325	Drowning	W65-W70.9, W73-W74.9	W65-W74.9	E910-E910.99	E91.0-E91.099
326	Fire, heat, and hot substances	X00-X06.9, X08-X19.9	X00-X19.9	E890-E899.09, E924-E924.99, E929.4	E89.0-E89.909, E92.4-E92.499, E92.94
327	Poisonings	E86.02-E86.99, J70.5, X40-X44.9, X46-X49.9, Y10-Y14.9, Y16-Y19.9	J70.5, X46-X48.9	E850.3-E858.99, E862-E869.99, E929.2	E85.6-E86.999
328	Poisoning by carbon monoxide	E86.2-E86.29, E86.8-E86.89, J70.5, X47-X47.9	J70.5, X47-X47.9	E862-E862.99, E868-E869.99	E86.2-E86.299, E86.8-E86.899, E86.990-E86.999
329	Poisoning by other means	E86.02-E86.19, E86.3-E86.7, E86.9-E86.99, X40-X44.9, X46-X46.9, X48-X49.9, Y10-Y14.9, Y16-Y19.9	X46-X46.9, X48-X48.9	E850.3-E858.99, E863-E866.99	E85.6-E86.199, E86.3-E86.709, E86.9-E86.99
330	Exposure to mechanical forces	W20-W38.9, W40-W43.9, W45.0-W45.2, W46-W46.2, W49-W52	W20-W38.9, W40-W43.9, W45.0-W45.2, W46-W52	E916-E922.99, E928.1-E928.7	E91.6-E92.299, E92.81-E92.87
331	Unintentional firearm injuries	W32-W34.9	W32-W34.9	E922-E922.99, E928.7	E92.2-E92.299, E92.87
332	Other exposure to mechanical forces	W20-W31.9, W35-W38.9, W40-W43.9, W45.0-W45.2, W46-W46.2, W49-W52	W20-W31.9, W35-W38.9, W40-W43.9, W45.0-W45.2, W46-W52	E916-E921.99, E928.1-E928.6	E91.6-E92.199, E92.81-E92.86
333	Adverse effects of medical treatment	D69.5-D69.59, D70.1-D70.2, D78-D78.89, D89.81-D89.813, E03.2, E06.4, E09-E09.9, E16.0, E23.1, E24.2, E27.3, E36-E36.8, E66.1, E87.0-E87.99, E89-E89.9, G21.0-G21.19, G24.0-G24.09, G25.1, G25.4, G25.6-G25.79, G62.0, G72.0, G93.7, G96.0, G96.11, G97-G97.9, H05.33-H05.339, H05.42-H05.53,	D69.5-D69.59, D70.1-D70.2, D78-D78.89, D89.81-D89.813, E03.2, E06.4, E09-E09.9, E16.0, E23.1, E24.2, E27.3, E36-E36.8, E66.1, E89-E89.9, G21.0-G25.79, G62.0-G97.9, H05.33-H05.339, H05.42-H05.53, H59-H59.89, H91.0-H91.09, H95-H95.9, I95.2-I97.9, J70-J70.4, J95-J95.9, K08.5-K08.59, K43-	244.0-244.1, 244.3, 251.3, 253.7, 279.5-279.53, 331.81, 333.92, 349-349.9, 357.6, 359.24, 379.6-379.63, 440.3-440.32, 457.0, 458.2-458.29, 518.6-518.7, 519.0-519.1, 525.6-525.79, 526.62-526.63, 530.86-530.87, 536.4-536.49, 539-539.9, 551.2-551.29, 552.2-552.29, 553.2-553.29, 564.2-564.4, 569.6-569.8, 579.3,	244.0-244.1, 244.3, 251.3, 253.7, 279.5-279.53, 331.81, 333.92, 349-349.9, 357.6-359.24, 379.6-379.63, 440.3-440.32, 457.0, 458.2-458.29, 518.6-519.1, 525.6-525.79, 526.62-526.63, 530.86-530.87, 536.4-536.49, 539-539.9, 551.2-551.29, 552.2-552.29, 553.2-553.29, 564.2-564.4, 569.6-569.8, 579.3, 595.82, 596.81-

		H59-H59.89, H91.0-H91.09, H95-H95.9, I95.2-I95.81, I97-I97.9, J70-J70.4, J95-J95.9, K08.5-K08.59, K43-K43.9, K52.0, K62.7, K68.11, K91-K91.9, K94-K95.89, L23.3, L27.0-L27.1, L56.0-L56.1, L64.0, L76-L76.82, M10.2-M10.29, M87.1-M87.19, M96-M96.9, N14-N14.4, N30.4-N30.41, N46.021, N46.121, N52.2-N52.39, N65-N65.1, N99-N99.9, P93-P93.8, P96.2, P96.5, R50.2-R50.83, Y40-Y84.9, Y88-Y88.3, Z21.0, Z42-Z43.0, Z43.8-Z43.9, Z48-Z48.9, Z51-Z51.9, Z88-Z88.9, Z92-Z94.0, Z94.6, Z94.8-Z94.9, Z96-Z96.49, Z96.6-Z97.2, Z97.8-Z99.12, Z99.3-Z99.9	K43.9, K52.0, K62.7, K68.11, K91-K91.9, K94-K95.89, L23.3, L27.0-L27.1, L56.0-L56.1, L64.0, L76-L76.82, M10.2-M10.29, M87.1-M87.19, M96-M96.9, N14-N14.4, N30.4-N30.41, N46.021-N46.121, N52.2-N52.39, N65-N65.1, N99-N99.9, P93-P93.8, P96.2-P96.5, R50.2-R50.83, Y40-Y84.9, Y88-Y88.3, Z21.0, Z42-Z51.9, Z88-Z94.0, Z94.6-Z99.9	595.82, 596.81-596.83, 598.2, 612-612.1, 779.4-779.5, 780.62-780.66, 995.89, E870-E876.9, E878-E879.9, E930-E949.9, V44-V45, V45.2-V45.4, V45.7, V45.77, V45.79-V45.8, V45.87-V45.89	596.83, 598.2, 612-612.1, 779.4-780.66, 995.89, E87.0-E87.99, E93.0-E94.99, V44-V45.89
334	Animal contact	W52.0-W62.9, W64-W64.9, X20-X29.9	W52.0-W64.9, X20-X29.9	E905-E906.99	E90.5-E90.699
335	Venomous animal contact		W52.3, X20-X29.9		E90.5-E90.599
336	Non-venomous animal contact	W52.0-W62.9, W64-W64.9, X20-X29.9	W52.0-W52.2, W52.4-W64.9	E905-E906.99	E90.6-E90.699
337	Foreign body	H02.81-H02.819, H44.6-H44.799, M60.2-M60.28, W44-W45, W45.3-W45.9, W75-W76.9, W78-W80.9, W83-W84.9	H02.81-H02.819, H44.6-H44.799, M60.2-M60.28, W44-W45, W45.3-W45.9, W75-W76.9, W78-W80.9, W83-W84.9	360.5-360.69, 374.86, 376.6, 709.4, 770.1-770.18, E911-E912.09, E913.8-E915.09	360.5-360.69, 374.86, 376.6, 709.4, 770.1-770.18, E91.1-E91.319, E91.38-E91.509
338	Pulmonary aspiration and foreign body in airway	W75-W76.9, W78-W80.9, W83-W84.9	W75-W76.9, W78-W80.9, W83-W84.9	770.1-770.18, E911-E912.09, E913.8-E913.99	770.1-770.18, E91.1-E91.319, E91.38-E91.399
339	Foreign body in eyes	H02.81-H02.819, H44.6-H44.799	H02.81-H02.819, H44.6-H44.799	360.5-360.69, 374.86, 376.6, E914-E914.09	360.5-360.69, 374.86, 376.6, E91.4-E91.409
340	Foreign body in other body part	M60.2-M60.28, W44-W45, W45.3-W45.9	M60.2-M60.28, W44-W45, W45.3-W45.9	709.4, E915-E915.09	709.4, E91.5-E91.509
341	Environmental heat and cold exposure	L55-L55.9, L58-L58.9, W88-W94.9, W97.9, W99-W99.9, X30-X32.9, X39-X39.9	L55-L55.9, L58-L58.9, W88-W99.9, X30-X32.9, X39-X39.9	E900-E902.99, E926-E926.99, E929.5	E90.0-E90.299, E92.6-E92.699, E92.95
342	Exposure to forces of nature	X33-X38.9	X33-X38.9	E907-E909.9	E90.7-E90.99
343	Other unintentional injuries	W39-W39.9, W77-W77.9, W81-W81.9, W85-W87.9, X50-X58.9	W39-W39.9, W77-W77.9, W81-W81.9, W85-W87.9, X50-X58.9	E903-E904.99, E913.2-E913.39, E923-E923.99, E927-E928.09, E928.8-E928.89	E90.3-E90.499, E91.32-E91.339, E92.3-E92.399, E92.5-E92.599, E92.7-E92.809, E92.88-E92.889
344	Self-harm and interpersonal violence	T74.2-U03, X60-X64.9, X66-Y08.9, Y35-Y38.9, Y87.0-Y87.2, Y89.0-Y89.1	T74.2-U03, X60-X64.9, X66-Y08.9, Y35-Y38.9, Y87.0-Y87.2, Y89.0-Y89.1	E950-E979.9, E990-E999.1	E95.0-E99.91

345	Self-harm	X60-X64.9, X66-X84.9, Y87.0	X60-X64.9, X66-X84.9, Y87.0	E950-E959	E95.0-E95.9
346	Self-harm by firearm	X72-X74.9	X72-X74.9	E955-E955.9	E95.5-E95.59
347	Self-harm by other specified means	X60-X64.9, X66-X71.9, X75-X84.9, Y87.0	X60-X64.9, X66-X71.9, X75-X84.9, Y87.0	E950-E954, E956-E959	E95.0-E95.4, E95.6-E95.9
348	Interpersonal violence	T74.2-T76.22, X85-Y08.9, Y87.1-Y87.2	T74.2-T76.22, X85-Y08.9, Y87.1-Y87.2	E960-E969	E96.0-E96.9
349	Physical violence by firearm	X93-X95.9	X93-X95.9	E965-E965.4	E96.5-E96.54
350	Physical violence by sharp object	X99-X99.9	X99-X99.9	E966	E96.6
351	Sexual violence	T74.2-T76.22, Y05-Y05.9	T74.2-T76.22, Y05-Y05.9	E960-E960.1	E96.0-E96.01
352	Physical violence by other means	X85-X92.9, X96-X98.9, Y00-Y04.9, Y06-Y08.9, Y87.1-Y87.2	X85-X92.9, X96-X98.9, Y00-Y04.9, Y06-Y08.9, Y87.1-Y87.2	E961-E964, E965.5-E965.9, E967-E969	E96.1-E96.4, E96.55-E96.59, E96.7-E96.9
353	Conflict and terrorism	U00-U03, Y36-Y38.9, Y89.1	U00-U03, Y36-Y38.9, Y89.1	E979-E979.9, E990-E999.1	E97.9-E99.91
354	Executions and police conflict	Y35-Y35.93, Y89.0	Y35-Y35.93, Y89.0	E970-E978	E97.0-E97.8
0	NOTE: This is a comprehensive mapping of ICD codes to GBD categories for Nonfatal Estimation based on Hospital Inpatient data. Not all causes use Hospital data. Detailed case definitions disease by disease are provided in the disease and injury specific write-ups. A small number of causes don't use ICD codes.				

HALE-50, HALE-70, HAQ and SDI in 2019

Country	HALE50	HAQI	SDI2019	HALE70
Afghanis	16.70	26.90	0.33	6.72
Bahrain	22.29	74.50	0.74	8.33
Djibouti	20.14	36.40	0.47	8.47
Egypt	19.52	57.00	0.63	7.83
Iran	23.73	70.90	0.70	10.05
Iraq	21.20	61.90	0.66	8.90
Jordan	24.18	70.80	0.73	10.31
Kuwait	25.72	82.60	0.85	11.40
Lebanon	22.51	81.10	0.73	9.41
Libya	22.87	63.10	0.74	10.05
Morocco	21.18	54.90	0.54	8.63
Oman	21.00	76.10	0.78	7.58

Palestin	19.15	35.60	0.48	7.85
Qatar	21.83	81.20	0.83	7.64
Saudi Ar	21.35	74.30	0.79	8.99
Somalia	16.94	16.10	0.08	7.18
Sudan	21.08	47.20	0.51	8.55
Syria	21.35	65.30	0.62	8.52
Tunisia	23.72	73.20	0.68	9.97
United A	20.73	61.20	0.88	8.42
Yemen	20.14	42.90	0.42	8.27

Age Standardized Estimates

1. **Male to female age standardized ratio of injury deaths in EMR:**

Between EMR countries, the highest overall age-standardized ratio of male to female deaths due to injury in 2019 was 4·2, found in Kuwait, with rates of 43·2 (UI 35·1-52·4) per 100, 000 in males and 10·3 (UI 8·7-12·5) per 100, 000 in females. Kuwait also had the highest male to female ratio for injury deaths among adults 50-64 at 4·9, with a rate of 157·2 (UI 118·1-203·6) for males and 32·3 (UI 25·0-42·2) for females and older adults (4·9), with a rate of 187·3 (UI 140·3-231·4) for males and 38·7 (UI 31·0-48·6) for females. Qatar had the highest male to female ratio for the elderly (3·1), with a rate of 214·0 (UI 136·4-307·4) for males and 60·4 (UI 41·4-86·1) for females per 100, 000· Afghanistan had the lowest reported male to female injury deaths ratio for adults (1·1) with a rate of 967·8 (UI 840·4-1,117·1) for males and 863·1 (UI 768·3-973·3) for females. Afghanistan also had the lowest ratio of 1·1 for older adults, with a rate of 1,320·1 (UI 1,191·4-1,458·6) for males and 1,238 (UI 1,131·7-1,365·4) per 100, 000. Syria had the lowest ratio for elderly (0·3), with a rate of 8,300·2 (UI 7,445·1-9,200·7) for male and 312406 (UI 28,495·5-34,390·0) for females per 100, 000. Syria reported the lowest ratio with a value of 1·1 and rates of 119·9 (UI 107·9-134·7) for males and 109·6 (UI 100·1-120·0) for females.

2. **Age standardized ratio of injury deaths by cause and country in EMR:**

The country with the highest transport injuries mortality rate in elderly was Oman, at 2,087·9 (UI 1,421·2-2,957·9), followed by Qatar at 1,661·4 (UI 1,026·7-2,761·4) and the UAE at 1,308·0 (UI 655·9-2,169·6) per 100,000. For older adults, the UAE had the highest death rates from transport injuries, at 278·7 (UI 131·0-456·1), followed by Oman at 275·2 (UI 192·4-395·6) and Saudi Arabia at 260·1 (UI 164·0-360·9) per 100,000· Saudi Arabia had the highest rate of injury deaths due to transport injuries in adults at 349·7 (UI 208·2-491·3), followed by the UAE at 237·5 (UI 115·1-400·8) and Egypt at 235·5 (UI 116·6-366·2) per 100,000.

Afghanistan had the highest death rates due to self-harm and interpersonal violence among all age groups. The rates were 10,800·5 (UI 9,828·0-11,889·3), 1,081·4 (UI 987·9-1,193·1), and 646·7 (UI 615·0-745·6) per 100,000 for elderly, older adults and adults respectively. Syria reported the second highest rates, with 8,736·1 (UI 7,938·9-9,635·2), 519·2 (UI 473·2-572·0), 278·9 (UI 253·5-307·4) per 100,000, respectively for the three groups. Yemen reported the third highest rate for elderly with, at 524·5 (UI 474·9-579·6 per 100,000. As for older adults and adults, Somalia was the third with rates of 519·2 (UI 473·2-572·0) and 196·5 (UI 128·1-292·6) per 100,000, respectively·

Oman had the highest mortality rates per 100,000 due to unintentional injuries at 3,397·9 (UI 1,672·2-5,028·1), followed by Somalia at 2,660·0 (UI 1,818·8-4,116·5) and Djibouti at 2,261·7 (UI 1,641·3-3,097·6). In older adults, Somalia took the lead in unintentional injuries at 323·7 (UI 220·2-578·5), followed by Djibouti at 192·8 (UI 141·5-268·8) per 100,000 and Saudi Arabia at 156·4 (UI 108·6-213·8) per 100, 000· Somalia also had the highest death rate in adults, at 257·5 (UI 167·5-469·7) and was followed by Saudi Arabia at 147·5 (UI 99·1-202·2) and Afghanistan at 134·9 (UI 95·3-189·1) per 100, 0000.

3. Age standardized Incidence and Prevalence

In 2019, the overall prevalence of injury in the EMR was 11,5316·7 (UI 106,340·6-124,897·7) per 100,000 for adults, 83,244·9 (UI 77,771·1-89,078·5) per 100,000 for older adults, and 279,140·3 (UI 262,886·7 – 295,693·4) for elderly in the EMR. The global burden was 116,612·9 (UI 109,929·5- 124,030·1) injuries per 100,000 for adults, 98529·6 (UI 92982·9- 104217·5) for older adults and 386,167·8 (UI 371,048·0- 402,697·3) for elderly.

The highest prevalence of injury in adults was reported in Afghanistan at 234,987·1 (UI 170,424·8 – 300,000) per 100,000, Iraq at 221,094·6 (UI 192,406·0- 243,465·1) and Lebanon at 162,402·8 (UI 120,353·4 – 229,532·4). Egypt, Jordan and Pakistan had the lowest injury prevalence in the EMR, at 75,114·5 (UI 70,623·1 – 80,795·3), 79,963·2 (UI 74,686·6 – 86,745·9) and 90,499·3 (UI 85,054·8 – 96,504·7) per 100,000, respectively.

Meanwhile, the highest prevalence of injury in older adults was in Afghanistan, Bahrain and Djibouti respectively, at 148,576·0 (UI 105,738·0- 200,000) per 100,000 153,152·6 (UI 142,824·2- 164,419·2) and 148,398·1 (UI 130,027·0- 162,696·7). The lowest injury prevalence was reported in Jordan at 199,728·2 (UI 189,736·9- 212,250·7) per 100,000, Yemen at 58,958·0 (UI 55,642·3-63,233·4), and UAE at 67,168·8 (UI 63,505·8- 72,056·8) per 100,000 population.

In the elderly population, the highest prevalence was also recorded in Afghanistan at 428,050·0 (354,942·3 – 500,000), followed by Syria at 470,525·7 (UI 441,135·8 – 493,371·8) and Iraq at 435,636·0 (UI 390,330·7- 472,439·4) injuries per 100,000 of population. The lowest prevalence was recorded in Egypt, Jordan and Bahrain at rates of 191,435·4 (UI 182,358·7 – 202,067·4), 199,728·2 (UI 189,736·9 212,250·7) and 218,585·8 (UI 207,054·4 – 233,013·2) injuries per 100,000, respectively.

The most prevalent type of injury in older adults was unintentional injuries at 62,963·2 (UI 58,287·9 – 69,031·2) and the least prevalent was transport injuries at 20,276·8 (UI 18,693·2- 22,040·4). In older adults, the most prevalent type of injury was unintentional injuries at 46,767·3 (UI 43,606·5– 50,706·4) while the least prevalent was traffic injuries at 17,595·1 (UI 16,328·0- 18,956·6). The most prevalent injuries in the elderly were also unintentional at 168,896·7 (UI 158,288·0- 182,254·2), while the least common injuries were transport injuries at 57,800·0 (UI 53,732·8- 62,322·3).

The number of new injury cases per 100,000 was 16,563·1 (UI 14,040·2- 19,423·3) in the EMR in 2019 for adults, 10,533·8 (UI 8,933·1- 12,422·3), and 45,200 (UI 38,634·3- 52,527·5) for elderly. Meanwhile, the global number of incident cases was 22,228·6 (UI 18,475·0- 26,752·4) in adults, 15,548·3 (UI 12,807·0- 18,784·9) in older adults and 88,456·1 (UI 71,192·5-110,755·7) per 100,000 in the elderly.

The highest reported incident cases of injury deaths among EMR adults in 2019 was found in Afghanistan at 54,017·9 (UI 42,782·8- 67,315·7), followed by Saudi Arabia at 28,457·0 (UI 24,213·8- 32,913·5) and Syria at 28,080·6 (UI 22,777·8- 34,174·3). The lowest incidences were recorded in Jordan, Egypt and Lebanon at 10,622·8 (UI 8,742·2-12,838·8), 10,953·7 (UI 9,149·7- 13,171·6) and 10,964·1 (UI 9,033·3-13,193·6) per 100,000 respectively.

Meanwhile, the leading countries in terms of incident cases for older adults were Afghanistan at a rate of 52,034·6 (UI 39,273·1- 68,493·7) per 100,000, Syria at 30,001·4 (UI 23,170·9- 38,764·3) and Saudi Arabia at 17,908·0 (UI 15,106·2- 21,471·9). The lowest reported incidences were in Lebanon, Jordan, and Bahrain at 5,922·9 (UI 5,028·2- 6,922·1), 6,073·1 (UI 5,196·8- 7,068·0) and 6,265·7 (UI 5,327·4- 7,283·2) per 100,000 respectively.

The highest incidence of injuries in the elderly was also in Afghanistan at 346,638·2 (UI 295,142·8- 393,302·5), followed by Syria at 345,374·3 (UI 292,062·6- 405,630·1) and Oman at 72,202·3 (UI 58,773·2- 88,123·8). The lowest incidence was in Egypt at a rate of 20,852·2 (UI 17,361·2·0-24,810·5), Jordan at 24,002·5 (UI 20,878·2- 27,523·2) and Sudan at 24,666·0 (UI 21,128·6- 28,618·1) per 100,000.

The most common types of incident injuries in the EMR were unintentional injuries for all three age groups at 10,460·3 (UI 8,563·1- 12,635·5), 5,864·3 (UI 4,865·0- 7,022·4), and 23,534·3 (UI 18,745·6-29,425·2) for adults, older adults and elderly, respectively. The least common type of incident injuries in adults was self-harm at 2,809·4 (UI 2,052·5-3,744·1) per 100,000 populations· In older adults and elderly, the least common incident

injuries were those due to transport injuries at rates of 2,065·2 (UI 1,320·5-2,939·0) and 5,131·4 (UI 3,575·1-7,165·6) respectively.

4. Age standardized ratio of YLLs by cause and country in the EMR:

For the adults, the highest YLLs were observed in Afghanistan at a rate of 28,983·3 (UI 25,644·6 – 32,791·5) per 100,000 population. It was followed by Somalia at 19,804·4 (UI 13,883·0 – 28,167·0) and Saudi Arabia at 16,734·9 (10920·0 - 22251·7). Meanwhile, Lebanon had the lowest ranking YLL rate at 2,587·3 (UI 1,962·7 – 3,424·0) for adults, followed by Bahrain at 2,682·9 (UI 1,967·0 – 3,631·3) and Jordan at 3,227·7 (UI 2489·5 - 4204·9).

For the older adults, the highest YLLs were observed in Afghanistan at a rate of 26,729·8 (UI 24,430·2– 29,297·5) per 100,000 population, followed by Somalia at 14,097·5 (UI 10,502·8– 19,674·0) and Syria with the rate 13,261·8 (11,958·9- 14,664·6). Concurrently, Lebanon had the lowest ranking YLL rate at 1,759·6 (UI 1,388·7– 2,265·9) for older adults, followed by Jordan at 2,404·1 (UI 1,898·1– 3,104·9) and Bahrain at 2,457·2 (UI 1,828·6 – 3,278·9).

For the elderly, the highest YLLs were observed in Afghanistan as well, with a rate of 100,360·3 (UI 91,196·9-110,756·1) per 100, 000 population, followed by the Syrian Arabic Republic at 81,211·3 (UI 73,712·3- 89,335·2) and Oman at 41,198·3 (UI 26,979·6- 55,684·1). Lebanon had the lowest YLLs for individuals aged 75 and more at 8,490·7 (5,570·7- 13,146·3). It was followed by Iraq and Jordan at rates of 9,061·5 (UI 6,504·6- 13,127·0) and 10,563·7 (UI 7,991·3- 13,892·8), respectively.

5. **Age standardized YLDs by cause and country in the EMR:**

The EMR age-standardized rate of YLD caused by injuries in 2019 was 4,296·0 (UI 3,145·0-5,768·2) per 100, 000 for adults; 3,004·3 (UI 2,215·0-3,971·2) for older adults' and 10,340·3 (UI 7,627·4-13,646·6) for the elderly per 100,000. The global rate in 2019 was higher in the population aged 50-74. The global rate of YLDs for adults was 4,268·6 (UI 3,044·5-5,753·1), and 3,702·4 (2,642·4-4,958·5) for older adults. The global YLD rate for the elderly was 17,519·6 (UI 12,425·1-23,813·1) per 100,000.

The leading cause of YLDs in the EMR was unintentional injuries for all age groups at a rate of 1,949·4 (UI 1,374·3-2,707·2) per 100, 000 for adults, 1,425·2 (UI 1,003·8-1,954·8) for older adults and 5,597·4 (UI 3,972·0-7,595·5) for the elderly.

For adults, the highest rate of YLDs was reported in Afghanistan at 12,798·0 (UI 6,261·7-25,157·6), followed by Iraq at 7,490·4 (5,516·0-9,995·0) and Saudi Arabia at 6,539·9 (4,527·8-9,130·1) per 100,000. Jordan had the lowest YLDs per 100, 000 with a rate of 2,473·4 (UI 1,730·1-3,434·2), followed by Egypt at 2,581·0 (UI 1,828·7-3,519·9) and Bahrain at 2,837·8 (UI 1,990·9-3,945·1).

For older adults, the highest rate of YLDs was reported in Afghanistan with a rate of 6,650·0 (UI 3,739·8-12,286·9), followed by Saudi Arabia at 5,233·8 (3,638·3- 7,202·1) and Syria at 4,883·1 (3,482·2- 6,642·4) per 100,000. Jordan had the lowest YLDs per 100, 000 with a rate of 1,865·7 (UI 1,307·6- 2,559·4), followed by Egypt at 1,945·0 (UI 1,365·4- 2,668·6) and Bahrain at 2,047·4 (UI 1,447·2- 2,814·3) per 100,000.

For the elderly, the highest rate of YLDs was reported in Syrian Arab Republic with a rate of 28,449·2 (20,699·1 – 37,699·7) per 100,000, followed by the Afghanistan at a rate of 22,120·3 (UI 14,961·7 – 33,696·7) and Saudi Arabia at 17,321·1 (UI 12,138·9 -23,703·5). Egypt, Jordan and Bahrain also had the lowest YLDs for this age population with rates at 6,498·8 (UI 4,588·0-8,883·3), 6,644·6 (UI 4,700·0-9,095·4) and 6,961·9 (UI 4,957·2-9,520·7) per 100,000, respectively.

6. Age standardized ratio of DALYs by cause and country in the EMR:

The country with the highest DALYs per 100,000 for adults in 2019 was reported to be Afghanistan, with 41,781·3 (UI 33,184·7-55,775·8), followed by Somalia with 24,140·3 (UI 18,122·7-32,878·5) and Saudi Arabia 23,274·9 (UI 16,977·6-29,366·2) with its relatively high SDI (0·80). Bahrain, Jordan and Kuwait had the lowest

DALYs rates, at 5,520·8 (UI 4,400·7-6,872·9), 5,701·2 (UI 4,590·1-6,985·3) and 6,707·8 (UI 5,489·5-8,217·9) per 100,000, respectively.

For older adults, Afghanistan had the highest DALYs per 100,000 in 2019 at a rate of 33,379·8 (UI 28,526·8-41,247·5), followed by Syria at 18,145·0 (UI 16,065·8-20,687·6) and Somalia at 17,326·6 (UI 13,410·5-22,817·5) per 100,000. For this population, Jordan had the lowest DALYs per 100,000 at 4,269·9 (UI 3,491·5-5,241·1), followed by the Bahrain at 4,504·7 (UI 3,613·1-5,542·5) and Lebanon at 4,918·1 (UI 3,760·6-6,823·5).

For the elderly, Afghanistan had the highest DALYs per 100,000 in 2019 at a rate of 122,480·7 (UI 108,282·6-142,908·9), followed by Syria at 109,660·6 (UI 98,196·2-122,649·3) and Oman at 53,595·7 (UI 38,624·3-68,629·1) per 100,000. For this population, Jordan had the lowest DALYs per 100,000 at 17,208·5 (UI 13,999·6-21,123·9), followed by Lebanon at 17,744·5 (UI 13,555·7-23,706·8) and Egypt at 18,057·8 (UI 14,080·0-22,785·3) per 100, 000.

7. Age Standardized Risk factors for injury-related deaths in the EMR:

The risk factor for injury-related deaths in adults were mostly linked to tobacco use in Somalia, with a death rate of 91·2 (UI 74·0-111·4) per 100,000, followed by low bone mineral density in Saudi Arabia at 73·6 (UI 44·0-101·2) and non-optimal temperature in the UAE, at 44·3 (UI 25·9-66·6) per 100,000.

The leading risk factor for injury-related deaths in older adults was associated with low bone mineral density, mostly in Saudi Arabia, followed by Oman and Somalia with death rates of 94·0 (UI 62·3-125·1), 72·5 (UI 5·1-96·9), and 67·5 (UI 47·7-92·8) per 100,000, respectively.

The most contributing risk factor associated with increased rates of injury death in the elderly were low bone mineral density, mostly in Oman with a rate of 1,925·1 (UI 1,045·1-2,947·2), followed by Qatar and Djibouti at 1,022·0 (UI 649·3-1,557·3) and 989·2 (UI 679·6-1,414·3) per 100,000, respectively.

Injury Mortality in the EMR

1. Deaths by Gender

EMR with a rate of 124·5 (UI 95·7-145·2) per 100,000 for males and 57·2 (UI 49·9-65·6) for females. Globally, the rate for males was 102·0 (UI 89·8-111·2) per 100,000 and 42·0 (UI 37·5-46·6) for females, with male to female ratio of around 2·4, higher than the ratio in the EMR. For older adults aged 70 and above, the ratio was 1·5, with rates of 256·9 (UI 221·7-293·8) and 173·6 (152·7-192·2) per 100,000 for males and females, respectively. Globally, rate of older adults injury death was 243·0 (UI 213·8- 264·4) per 100, 000 for males and 182·7 (UI 153·1-203·6) for females, with a ratio of 1·3, similar but slightly lower than the EMR-specific rate.

Across EMR countries, the highest ratio of male to female deaths due to injury among adults aged 50-69 in 2019 was 4·7, found in Kuwait, with rates of 51·4 (UI 39·5-65·8) per 100, 000 deaths in males and 10·8 (UI 8·8-13·8) in females. Afghanistan reported the lowest ratio with a value of 1·1 and rates of 317·4 (UI 276·9-360·6) for males and 291·8 (UI 261·7-324·9) for females. Kuwait also had the highest ratio for older adults aged 70 and above with a value of 2·9 and rate of 199·8 (UI 154·6-241·9) deaths per 100,000 in males and of 67·8 (52·8-82·0) in females. Afghanistan also had the lowest ratio, 1·1, with the rates 1,185·6 (1086·9-1292·8) for males and 1,041·2 (953·1-1141·3) for females.

2. Death by Country

For the strata of ages 50-69 years, Afghanistan had the highest death rate with a rate of injury deaths at 304·2 (UI 272·4- 340·3), followed by Somalia at 222·4 (UI 161·8-303·7) and Saudi Arabia at 174·0 (UI 114·7-221·3). The lowest reported rate for this group was in Lebanon, at a rate of 28·5 (UI 22·6-36·1), followed by Bahrain at 29·3 (UI 22·2-38·7) and Kuwait at 34·6 (27·3-43·0) per 100, 000.

The country with the highest rate of death in the older adults population due to transport injuries was Oman with a rate of 191·0 (143·4-287·7) per 100, 000 followed by the UAE at 151·6 (78·2-242·4) and Qatar at 138·8 (98·3-

188·1). For adults, Saudi Arabia had the highest death rates from transport injury, at 117·5 (UI 71·1-153·4), followed by Egypt 81·3 (UI 41·4-115·7) and Yemen at 80·7 (UI 48·6-115·0).

The countries with the highest death rate in the older adults due to self-harm and interpersonal violence were Afghanistan with 963·1 (UI 876·2-1061), followed by the Syria with 593·6 (UI 539·9- 653·5), and Somalia with 83·3 (UI 60·1-111·5) deaths per 100, 000. For adults, the countries with the highest rates of death per 100, 000 were Afghanistan at 224·5 (UI 205·2-247·3), the Syria at 105·2 (UI 95·8-116·0) and Somalia at 67·8 (46·7-94·9).

In the older adults aged 70 and above, Somalia had the highest rate of deaths per 100,000 due to unintentional injuries at 245·3 (UI 180·0-394·0), followed by Djibouti at 178·3 (UI 144·6-231·1) and Saudi Arabia at 137·2 (UI 113·2-161·5)· In adults, Somalia lead in unintentional injuries at a rate of 94·3 (62·8-168·7).

3. Injury Prevalence in the EMR

In 2019, the overall prevalence of injury was 38,720·2 (UI 35,730·1-41,804·5) per 100,000 for adults aged 50-69, and 46,324·2 (UI 43,584·4-49,214·1) for older adults aged 70 and above in the EMR. The global burden was 39,989·6 (UI 37,852·2-42,221·2) injuries per 100,000 for adults 50-69 and 61,219·5 (UI 58,413·3-64,271·5) for older adults 70 and above.

The highest prevalence of injury in adults aged 50-69, was reported in Afghanistan at 79,684·1 (UI 57,261·8-100,000) per 100,000, Iraq at 73,840·6 (UI 64,422·0-80,997·1) and Palestine 64,156·0 (59120·5-71193·4)· Egypt, Jordan and Pakistan had the lowest injury prevalence in the EMR, at 25,350·3 (UI 23,937·4-27,100·4), 26,799·2 (UI 25,177·2-28,890·8) and 30,524·6 (UI 28,895·9-32,273·4) per 100,000, respectively.

In older adults aged 70 and above, the highest prevalence was also recorded in Afghanistan at 77,438·0 (57,830·0-100,000), followed by Djibouti with 47,979·0 (UI 45,782·3-50,558·7) injuries per 100,000 of population. The lowest prevalence was recorded in Yemen, the United Arab Emirates and Tunisia with 42,201·2 (UI 40,217·9-44,571·5), 43,231·6 (UI 40,982·8-46,115·5) and 48,106·2 (UI 43,501·8-53,848·0) injuries per 100,000, respectively.

The most prevalent type of injury in adults aged 50-69 was unintentional injuries at 21,123·5 (UI 19,568·4-23,165·2) and the least prevalent was injuries executions and police conflicts at 140·1 (UI 85·3-221·7). The most common injuries by country were due to unintentional injuries in Iraq (34,542·0 (UI 31,704·2-37,745·1)) and Saudi Arabia (52,283·5 (UI 47,393·2-58,934·9)) and conflict and terrorism in Afghanistan (61,440·8 (UI 28,079·8-95,544·7)).

The most and least prevalent injuries in older adults aged 70 and above were also unintentional injuries and those due to executions and police conflicts, at 26,485·4 (UI 24,888·5-28,563·4) and 110·0 (UI 72·6-161·1) per 100,000 respectively· The most prevalent injuries per country were unintentional injuries in Saudi Arabia (70,130·0 (UI 65,072·7-77,106·5)), followed by conflict and terrorism in Afghanistan (52,469·0 (UI 23,619·8-91,469·0)) and the Syria (45,144·9 (UI 31,501·7-61,362·3)) per 100,000·

4. Injury Incidence in the EMR

The number of new injury cases per 100,000 was 5,544·1 per year (UI 4,923·0-6,179·1) in the EMR in 2019 for adults aged 50-69 and 6,655·8 (UI 5,985·2-7,382·8) for older adults aged 70 and above. Meanwhile, the global number of new cases was 7,423·3 (UI 6,538·9-8,410·3) for adults aged 50-69 and 10,953·8 (UI 9,677·4-12,467·5) per 100,000 for older adults aged 70 and above.

The highest number of new cases among EMR adults aged 50-69 in 2019 was found in Afghanistan at 17,942·4 UI (14,217·4-20,983·6), the Syria at 9,571·8 (UI 8,299·8-11,286·9) and Saudi Arabia at 9,639·4 (UI 8,655·4-10,692·9). The lowest incidences were in Jordan, Lebanon and Egypt at 3,569·6 (UI 3,078·6-4,116·9), 3,579·9 (UI 3,102·1-4,090·9) and 3,629·3 (UI 3,176·0-4,119·4) per 100,000 respectively.
The highest incidence of injuries in older adults aged 70 and above was found in Afghanistan at 39,588·0 (UI 33,224·0-46,685·2), followed by the Syrian Arab Republic at 30,865·0 (UI 25,903·2-36,222·1) and Saudi Arabia at 10,282·2 (UI 9,153·4-11,505·7). The lowest incidence was found in Bahrain at 3,814 (UI 3,461·0-4,182·1), Jordan at 3,537·5 (UI 3,203·3-3,891·9) and Egypt at 3,531·9 (UI 3,109·1-3,976·8) per 100,000 (Table 1, 2).

The most common types of injuries for EMR in 2019 were unintentional injuries at 3,468·6 (UI 2,972·2-3,984·1) for adults aged 50-69, and transport injuries for older adults aged 70 and above at 986·6 (UI 732·9-1,253·9)· The most incident injury type per country was unintentional injuries in Saudi Arabia, Qatar and Iraq for adults at 7,986·2 (UI 7,084·8-9,005·3), 4,985·5 (UI 4,192·0-5,862·0) and 4,933·9 (UI 4,138·1-5,723·9) per 100,000, respectively. Unintentional injuries were also the most common type of new injuries in older adults aged 70 and above in Saudi Arabia, Oman and Somalia at 8,793·8 (UI 7,719·3-9,978·1), 6,359·0 (UI 5,589·5-7,187·1) and 5,013·3 (UI 4,305·2-5,909·8), respectively.

5. YLDs in the EMR

In 2019, the rate of Years Lived with Disability (YLDs) caused by injuries was 1,439 (UI 1,057·2-1,918·9) per 100, 000 for adults aged 50-69 and 1,679·2 (UI 1,239·3-2,228·5) for older adults aged 70 and above in the EMR. The global rate in 2019 is slightly higher at 1,469·4 (1,049·3-1,983·6) for those aged 50-69 and 2,410·5 (UI 1,723·7-3,250·1) per 100, 000 (Table 1, 2).

The leading cause of YLDs in the EMR was unintentional injuries at a rate of 653·3 (UI 460·9-910·1) per 100, 000 for those aged 50-69 and 836·1 (UI 596·7-1,139·5) for ages 70 and above. Globally, unintentional injuries were also the leading cause of YLDs in 2019, at a rate of 850·5 (UI 592·8-1,172·5) for those aged 50-69 and 1,755·9 (UI 1,247·9-2,393·8) per 100, 000 for those 70 and above.

For those aged 50-69, the highest rate of YLDs was reported in Afghanistan with a rate of 4,346·8 (UI 2,128·8,672·3), followed by Iraq at 2,479·8 (1,834·5-3,296·4) and the Syria at 2,184·2 (1,509·3-3,031·1) per 100,000. Jordan had the lowest YLDs per 100, 000 with a rate of 827·8 (UI 585·1-1,146·9), followed by Egypt at 869·2 (UI 618·5-1,182·9) and Bahrain at 946·8 (UI 670·6-1,313·0).

For those aged 70 and above, the highest rate of YLDs was reported in Afghanistan with a rate of 3,476·2 (2,134·7-5,915·7) per 100,000, followed by Syria at a rate of 3,291·3 (UI 2,384·0-4,422·8) and Saudi Arabia at 2,931 (UI 2,055·1-4,001·3). Jordan, Egypt and Bahrain had the lowest YLDs for this age population with rates at 1049·2 (743·1-1,433·2), 1,080 (UI 766·7-1,478·1) and 1,092·8 (UI 783·1-1,500·7) per 100,000, respectively.

6. YLLs in th EMR

In the EMR, the rate of injury related Years of Life Lost (YLLs) in 2019 was 2,866·1 (UI 2,344·1-3,298·6) per 100, 000 for adults aged 50-69 and 3,054·4 (UI 2,691·2- 3,389·5) for older adults aged 70 and above. The regional rate is higher than the global rate for both populations, with a rate of 2,173·6 (UI 1,959·7- 2,349·0) for those aged 50-69 and 2,680·2 (UI 2,361·4- 2,907·6) for those aged 70 and above globally. YLLs was also consistently higher in the older adults aged 70 and above compared to adults aged 50-69 (Tables 1, 2).

The leading causes of YLLs were transport injuries for 50 to 69 age group at a rate of 1,394·1 (UI 1,012·4-1679·0) per 100,000, and unintentional injuries for the 70 and above age group at 1,177·0 (UI 998·1-1,408·7) per 100,000. In contrast, the leading global cause of injury YLLs was unintentional injuries for both age groups, at 836·1 (UI 719·9-918·4) per 100, 000 population for adults aged 50-69, and 1,754·9 (UI 1,490·1-1,930·6) for older adults aged 70 and above.

For the age group 50 to 69, the highest YLLs were observed in Afghanistan at a rate of 9,608·7 (UI 8571·2-10,810·3) per 100, 000 population, followed by Somalia at 6,700·9 (UI 4,850·8-9,220·8) and Saudi Arabia with the rate 5,596·7 (UI 3,704·6-7,156·2). While, Lebanon had the lowest ranking YLL rate at 866·6 (UI 688·7-1107·3) for ages 50-69, followed by Bahrain at 916·4 (UI 695·2- 1,214·3) and Jordan at 1,095·0 (UI 866·8-1,397·1) per 100,000.

For the age group 70 and above, the highest YLLs were observed in Afghanistan, with a rate of 15,838·7 (UI 14,531·9- 17,322·8) per 100, 000 population, followed by the Syria at 9,507·3 (UI 8,642·4-10,426·6) and Somalia at 6,827 (UI 5,301·1-9,247·1). Lebanon had the lowest YLLs for individuals aged 70 and more at 1,089·7 (869·9-1,480·6). It was followed by Jordan and Bahrain at rates of 1,356·1 (UI 1,113·8-1,691·3) and 1,565·7 (UI 1,260·7-1,924·1), respectively (Table 1, 2).
